# The Immunome of Colon Cancer: Functional *In Silico* Analysis of Antigenic Proteins Deduced from IgG Microarray Profiling

**DOI:** 10.1016/j.gpb.2017.10.002

**Published:** 2018-03-02

**Authors:** Johana A. Luna Coronell, Khulan Sergelen, Philipp Hofer, István Gyurján, Stefanie Brezina, Peter Hettegger, Gernot Leeb, Karl Mach, Andrea Gsur, Andreas Weinhäusel

**Affiliations:** 1 Molecular Diagnostics, AIT – Austrian Institute of Technology, A-1190 Vienna, Austria; 2 Department of Medicine I, Institute of Cancer Research, Comprehensive Cancer Center, Medical University Vienna, A-1090 Vienna, Austria; 3 Hospital Oberpullendorf, A-7350, Oberpullendorf, Austria

**Keywords:** Autoantibody tumor biomarker, Cancer immunology, Colorectal cancer, Immunomics, Protein microarray

## Abstract

Characterization of the colon cancer immunome and its autoantibody signature from differentially-reactive antigens (DIRAGs) could provide insights into aberrant cellular mechanisms or enriched networks associated with diseases. The purpose of this study was to characterize the antibody profile of plasma samples from 32 **colorectal cancer** (CRC) patients and 32 controls using proteins isolated from 15,417 human cDNA expression clones on microarrays. 671 unique DIRAGs were identified and 632 were more highly reactive in CRC samples. Bioinformatics analyses reveal that compared to control samples, the immunoproteomic IgG profiling of CRC samples is mainly associated with cell death, survival, and proliferation pathways, especially proteins involved in EIF2 and mTOR signaling. Ribosomal proteins (*e.g.*, RPL7, RPL22, and RPL27A) and CRC-related genes such as *APC*, *AXIN1*, *E2F4*, *MSH2*, *PMS2*, and *TP53* were highly enriched. In addition, differential pathways were observed between the CRC and control samples. Furthermore, 103 DIRAGs were reported in the SEREX antigen database, demonstrating our ability to identify known and new reactive antigens. We also found an overlap of 7 antigens with 48 “CRC genes.” These data indicate that **immunomics** profiling on **protein microarrays** is able to reveal the complexity of immune responses in cancerous diseases and faithfully reflects the underlying pathology.

## Introduction

Colorectal cancer (CRC) is reported worldwide as the second most common cancer in women and third in men, which makes it a leading cause of cancer-associated mortality in developed countries [1], [2]. Various screening methods for CRC are available, such as fecal occult blood tests (FOBT), colonoscopy, and flexible sigmoidoscopy [3]. Implementation of nationwide screening programs, and minimal invasive and early diagnostic methods could help to reduce the high mortality rate of CRC. Early diagnostic methods would enable prompt detection of cancer at early stages, which is essential for therapeutic success and a higher patient survival rate. Therefore, discovery, and identification of sensitive as well as specific markers that could be exploited at the earliest possible stage is needed. Ideally, the identification of biomarkers shall be established with easy sample access [4] from body fluids like serum, plasma or saliva in a minimally invasive manner, which are generally preferred than undergoing colonoscopy.

In cancer, altered protein expression during neoplastic transformation and tumor progression can elicit immune responses and induce the formation of tumor autoantibodies [5]. Besides the involvement in inhibiting tumor growth, immune responses could also promote tumor growth through a process called immunoediting consisting of elimination, equilibrium, and escape phases [6], [7]. Immunoediting may affect the composition and quantity of circulating antibodies. The reactivity of these antibodies toward recognized or unrecognized tumor-associated antigens (TAAs) can be affected by multiple factors related to cancer growth, such as aberrant expression of differentiation genes, accumulation of mutations, inaccurate post-translational modifications, alternative splicing, as well as deregulated necrotic or apoptotic processes [8], [9]. These TAAs usually have key functions in tumorigenesis, for instance, regulation of cell proliferation and cycle, differentiation, and apoptosis [10], [11]. Antibodies are very stable and can be detected months or even years before a clinical cancer diagnosis [12], which makes it possible to determine the differentially-reactive antigens (DIRAGs) among patients as well as relative to control samples by analyzing the immunome (antibody profile) [13], [14]. Therefore, autoantibodies could be used as a serologic tool for early diagnosis of cancer.

Autoantibody signatures for several cancer types have been reported, including colon cancer, prostate cancer, breast cancer, liver cancer, ovarian cancer, renal cancer, head and neck cancer, esophageal cancer, lymphoma, and leukemia [15], [16], [17], [18], [19], [20], [21], [22]. Autoantibodies in cancer can be identified using various methods, such as phage display [12], [23], serological analysis of recombinant cDNA expression libraries (SEREX) [15], [24], and serological proteomics analysis (SERPA, also known as Proteomex) [25], [26], [27]. However, these techniques require complex steps [28], [29]. There exists significance and need for identifying new protein biomarkers in CRC, as reviewed by us [3] and lately by Coughlin and Murray [30].

Protein arrays, which comprise recombinant proteins, protein fractions, or purified proteins, offer a potent tool for both definition and identification of immune profiles [31]. Proteins included in the arrays are known, which are printed with a comparable concentration in a highly-multiplex manner. Therefore, there exists no bias in identification of biomarkers with great sensitivity [32]. Additionally, high-density protein arrays increase the chance of discovering novel autoantibodies against low abundance proteins while also allowing testing of thousands of proteins simultaneously [33]. Thus, detection of diagnostic autoantibody signatures by testing patient samples from, *e.g.*, cancer patients versus control samples, can be conducted in a cost-effective manner [3], [34]. A review on protein-based approaches for biomarker discovery was recently done by Huang and Zhu [35].

In this study, we have produced and tested protein microarrays from 15,417 human cDNA expression clones presenting 6369 unique human proteins for the identification of DIRAGs [36]. Our previous work has demonstrated that using purified IgG does avoid artifacts caused by the matrix of serum or plasma samples, and is thus an ideal way to analyze DIRAG profiles [37]. Thus IgG derived from heparin-plasma of 32 CRC patients and 32 controls was used in the current study. As a result, we show that biological profiles can be illustrated via antibody profiling.

## Results

### IgG profiling on protein-microarrays

To identify DIRAGs from IgG profiling, we performed the immunoprofiling of CRC and control samples using our in-house protein microarray as previously described [36]. All plasma samples used were collected in the ongoing molecular epidemiology “Colorectal Cancer Study of Austria” (CORSA), targeted to inhabitants of the Austrian province Burgenland aged 40–80 years, as described in the Material and Methods section. After the data were normalized using distance weighted discrimination (DWD), we used *t*-test (*P* = 0.01 as cut-off) to evaluate the differences in antibody profiles between the CRC and control samples. Consequently, 671 unique antigenic proteins were identified as DIRAGs based on the median fold-change between classes. Among them, 632 antigenic proteins were found to be higher reactive in CRC samples, whereas 31 antigenic proteins were more reactive in the control samples. However, we also found that 8 antigenic proteins exhibited unclear immunoreactivity, *i.e.*, two different antigen clones expressing the same proteins were found to be significantly different in immunoreactivity but in opposite directions (one clone with increased immunoreactivity and the other with reduced immunoreactivity) between CRC and control samples. The list of significant antigenic proteins can be found in Table S1. The technical performance and reliability of the protein array analysis is provided as described in the methods and shown in Figure S1.

**Figure 1:**
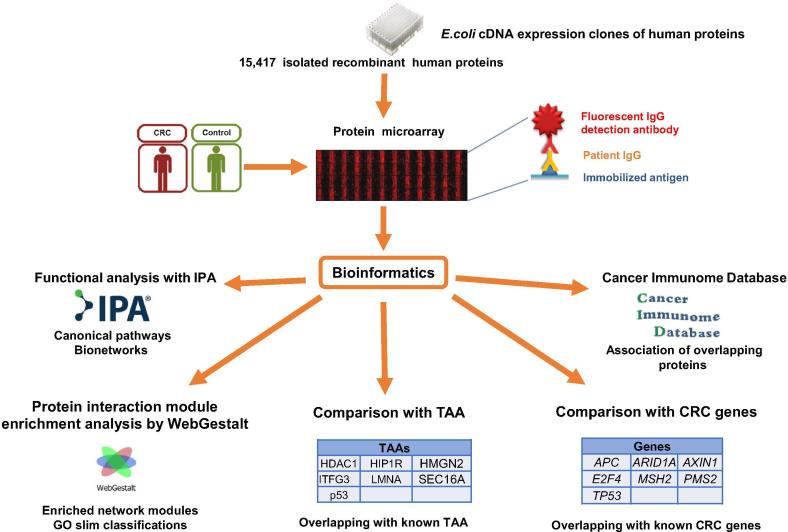
**Procedure overview** The described procedure exemplifies the methodology used in this study. An expression library consisting of 15,417 cDNA clones was used to produce recombinant human proteins. The recombinant proteins were isolated and used for printing protein microarrays. IgG was isolated from a total of 64 samples (32 CRC samples and 32 healthy control samples) and tested on the protein microarrays. Bioinformatics analyses (*t*-tests) were performed to identify the DIRAGs between the groups of arrays. Subsequently, the list of DIRAGs were subjected to functional analysis with IPA, hierarchical protein interaction module enrichment analysis with WebGestalt, association of overlapping proteins with the Cancer Immunome Database analysis, and analysis of overlap with known CRC and TAAs. CRC, colorectal cancer; TAA, tumor-associated antigen; GO, Gene Ontology.

These 671 DIRAGs were subjected to bioinformatics analyses as outlined in Figure 1.

### Functional analysis for associated pathways and networks

To identify the associated canonical pathways and network functions, we then performed functional analysis using Ingenuity Pathway Analysis (IPA, Ingenuity® Systems, www.ingenuity.com) [38]. As shown in Table S2, DIRAGs were involved in 50 canonical pathways (*P <* 0.01; −log *P* value >2). The top 5 pathways include the eukaryotic initiation factor 2 (EIF2) signaling pathway, mTOR signaling, growth hormone signaling, virus entry via endocytic pathways, and 14-3-3-mediated signaling (Table 1).

**Table 1:** **Top 5 pathways enriched with DIRAGs**

**Pathway**	**-Log (*P* value)**	**Ratio**	**Proteins**
EIF2 signaling	5.39	0.162	PABPC1, PIK3C2B, RPL22, RPL27A, RPL37A, RPS19, PDPK1, PPP1R15A, RPS17/RPS17L, EIF4G1, RPL7, RPS7, EIF3G, EIF3F, RPS27, EIF4G2, RPL28, RPL36AL, RPL19, RPS25, PIK3CD, PIK3R2, RPS10, RPL18

mTOR signaling	4.63	0.150	PIK3C2B, ULK1, DDIT4, RPS19, PDPK1, RPS17/RPS17L, EIF4G1, PRKCZ, EIF3G, RPS7, DGKZ, EIF3F, RPS27, EIF4G2, PRKCD, TSC2, RPS6KB2, RPTOR, RPS25, PRKCH, PIK3CD, PIK3R2, RPS10

Growth hormone signaling	4.41	0.226	PIK3C2B, PRKCD, RPS6KB2, PLCG1, PDPK1, PRKCH, PIK3CD, STAT3, PIK3R2, STAT1, ELK1, PRKCZ

Virus entry via endocytic pathway	3.71	0.183	PIK3C2B, FLNB, AP1G2, HLA-C, HLA-A, PRKCD, CLTA, HLA-B, PLCG1, PIK3CD, PRKCH, PIK3R2, PRKCZ

14-3-3-mediated signaling	3.45	0.158	PIK3C2B, TUBB3, YWHAE, PDIA3, YWHAZ, PLCG1, VIM, PRKCZ, PRKCD, TSC2, PIK3CD, PRKCH, PIK3R2, ELK1, PDCD6IP

*Note*: The ratio is the number of proteins in a given pathway that meet the cutoff criteria (*P* < 0.01), divided by the total number of proteins that make up that pathway. The complete list of 50 pathways can be found in Table S2.

IPA analysis revealed that the EIF2 signaling pathway was the most overrepresented canonical pathway between CRC and control samples (*P* = 4 × 10^−6^). A total of 24 proteins were represented in the EIF2 signaling pathway, including three proteins from the phosphoinositide 3-kinase (PI3K) family, namely phosphatidylinositol-4,5-bisphosphate 3-kinase catalytic subunit δ (PIK3CD), PIK3C type 2 β (PIK3C2B), and PI3K regulatory subunit 2 β (PIK3R2). PI3Ks are involved in signaling pathways such as cell motility, cell migration, vesicle transport, and apoptosis [39].

To identify interactions at the molecular level between the DIRAGs found (Table S1) and how they might work together, we then analyzed mechanistic bionetworks using IPA. As shown in Table 2, Table 3 out of the 5 bionetworks found are related to cell death and survival, with one related to cancer as well. In addition, 3 bionetworks are involved in cellular growth and proliferation (Table 2). The detailed list of related DIRAGs can be found in Table S3.

**Table 2:** **Top 5 associated network functions obtained with IPA**

**Associated network functions**	**Score**	**No. of DIRAGs found**
Cell death and survival, cell cycle, cellular growth and proliferation	40	35
Cellular movement, cellular growth and proliferation, cell cycle	11	16
Cell cycle, cellular development, cellular growth and proliferation	11	18
Cell death and survival, cell cycle, cellular development	10	17
Cell death and survival, cancer, reproductive system disease	8	15

*Note*: The score indicates the likelihood of the focus genes in a network being found together due to random chance and is used to rank networks according to their degree of relevance to the network eligible molecules in a dataset, based on the connectivity of the molecules in a given network. The score is calculated with the right-tailed Fisher's Exact test. The maximum network size is set at 35 by default.

### Protein interaction enrichment analysis with WebGestalt

Comparison of the DIRAGs with the protein list from the annotated genes presented in the UniPEx library in pre-defined functional categories was performed for a hierarchical protein interaction module enrichment analysis. The hierarchical relationship of the enriched phenotype terms can be observed in the directed acyclic graph (DAG) found in Figure S2.

**Supplementary Figure S3:**
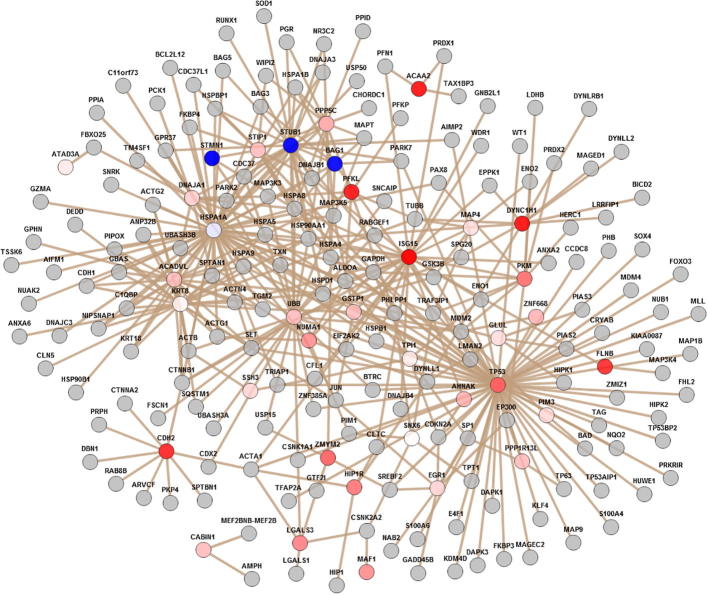
**Node-link diagram visualization of DIRAGs enriched in Module 1**Visualization of higher antigenic reactivity (up-regulated, colored from white to red) and low-antigenic reactivity (down-regulated, colored from blue to white) DIRAGs in CRC samples in comparison with control samples (in the center and their direct neighbors (at the edge) with WebGestalt. Enrichment analysis was performed using the hypergeometric test, and the Benjamini–Hochberg procedure was used for multiple test adjustment. A minimum number of two genes for a category was used as cut-off, and pathways significance level was set at *P* = 0.01 (*t*-test).

**Supplementary Figure S4:**
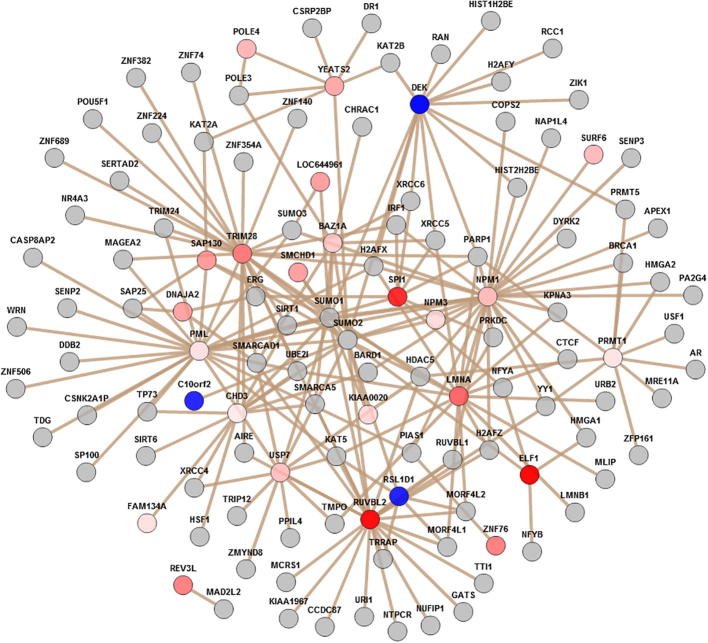
**Node-link diagram visualization of DIRAGs enriched in Module 2**Visualization of higher antigenic reactivity (up-regulated, colored from white to red) and low-antigenic reactivity (down-regulated, colored from blue to white) DIRAGs in CRC samples in comparison with control samples (in the center and their direct neighbors (at the edge) with WebGestalt. Enrichment analysis was performed using the hypergeometric test, and the Benjamini–Hochberg procedure was used for multiple test adjustment. A minimum number of two genes for a category was used as cut-off, and pathways significance level was set at *P* = 0.01 (*t*-test).

Among the 19 enriched network modules, three modules are found to contain 14-36 proteins. These include Module 1 (36 proteins), Module 2 (26 proteins), and Module 3 (14 proteins). As shown in Figure S3, some higher antigenic reactive proteins are overexpressed (up-regulated, in red) in Module 1. These include proteins involved in translation factors, *e.g.*, ISG15 ubiquitin-like modifier (ISG15), as well as transport and cytoskeleton, *e.g.*, dynein cytoplasmic 1 heavy chain 1 (DYNC1H1) and filamin B (FLNB). Proteins in Module 2 are mostly transcription factors, or proteins associated with double-strand break repair and DNA binding (Figure S4).

**Figure 2:**
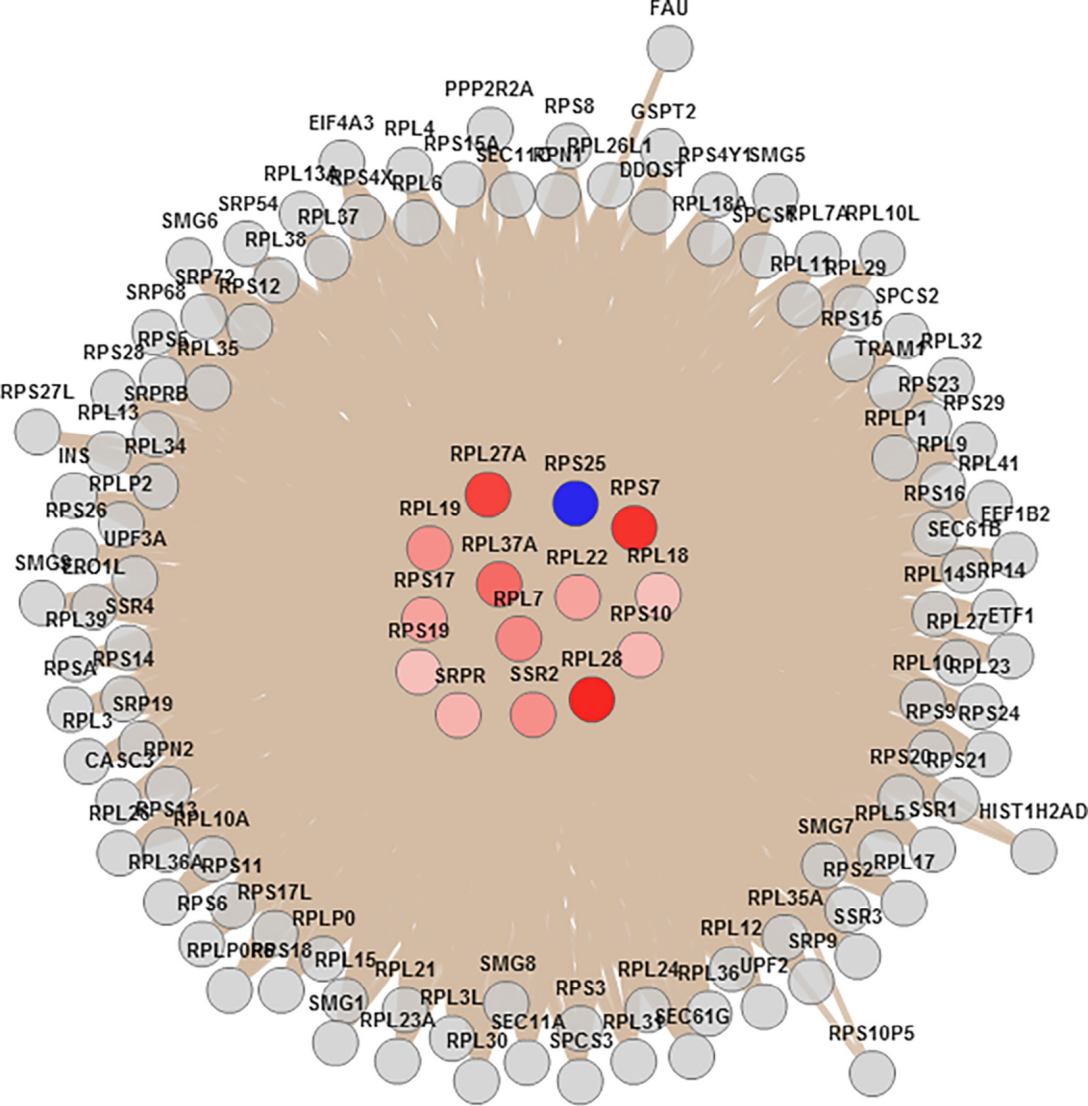
**Node-link diagram visualization of DIRAG-enriched Module 3** Visualization of higher antigenic reactivity (up-regulated, colored from white to red) and low-antigenic reactivity (down-regulated, colored from blue to white) DIRAGs in CRC samples in comparison with control samples (in the center) and their direct neighbors (at the edge) was obtained using the protein interaction enrichment analysis in WebGestalt. Enrichment analysis was performed using the hypergeometric test, and the Benjamini–Hochberg procedure for multiple test adjustment (*P =* 0.01). CRC, colorectal cancer; DIRAG, differentially-reactive antigen.

Figure 2 shows the node-link diagram for Module_3, which contains 12 ribosomal proteins including 5 L ribosomal proteins (RPLs), *i.e.*, RPL7, RPL18, RPL19, RPL22, RPL27A, RPL28, and RPL37A, and 5 ribosomal protein S, *i.e.*, RPS7, RPS10, RPS17, RPS19, and RPS25. In addition, signal recognition particle receptor (SRPR) and signal sequence receptor subunit 2 (SSR2) in the endoplasmic reticulum were found in Module 3 as well.

Of note, we found that ribosomal proteins were also enriched in the EIF2 signaling pathway obtained with IPA (Table 1). Therefore, we compared the proteins from Module_3 and the proteins from the EIF2 signaling pathway. We thus found an overlap of 12 ribosomal proteins, including RPL7, RPL18, RPL19, RPL22, RPL27A, RPL36AL, RPL37A, RPS7, RPS10, RPS17, RPS19, and RPS25. This result indicates that complex cellular structures (especially ribosomes) are a frequent target of autoantibodies.

**Figure 3:**
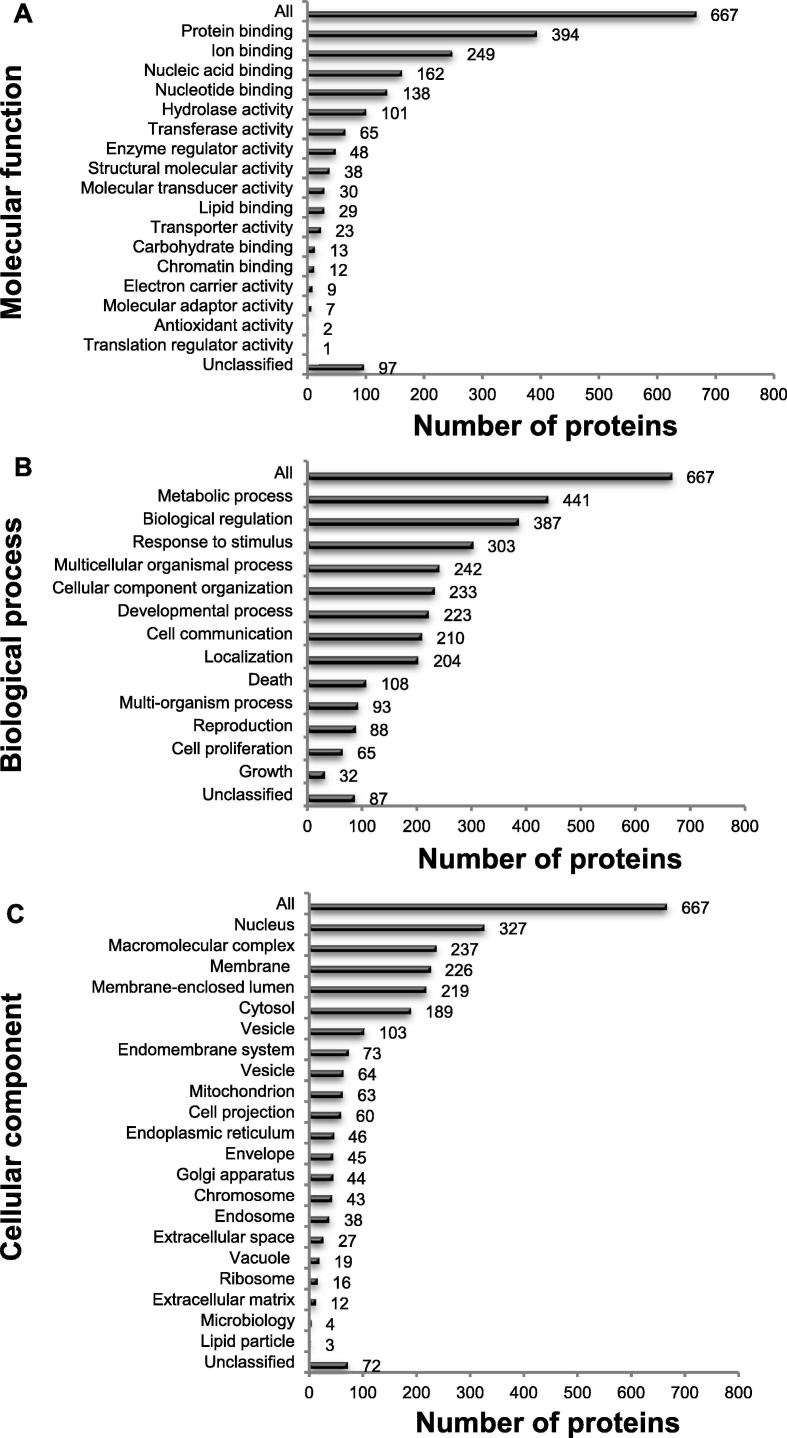
**GO Slim classification analysis of the 671 DIRAGs identified** Histogram of functional annotations of DIRAGs in CRC samples in comparison with control samples (*P =* 0.01) was generated based on the WebGestalt derived GO slim charts in the three GO functional categories. **A.** Molecular function. **B.** Biological process. **C.** Cellular component. More than half of the proteins are nuclear proteins. DIRAG, differentially-reactive antigens; CRC, colorectal cancer; GO, Gene Ontology.

To gain further understanding of the biological meaning of the DIRAGs, we performed Gene Ontology (GO) slim classifications [40]. Molecular function analysis indicated that DIRAGs are predominantly involved in binding functions (394 of 671), including protein, ion, nucleic acid and nucleotide acid binding (Figure 3**A**). The biological process analysis showed that 66% of DIRAGs were found in metabolic processes (441 proteins), while 58% were involved in biological regulation (387 proteins) (Figure 3B). Furthermore, cellular component analysis revealed that the classified proteins were mainly found in nuclear components (327 DIRAGs), macromolecular complexes (237 DIRAGs), membrane function (226 DIRAGs), membrane enclose lumen (219 DIRAGs), and cytosol (189 DIRAGs) (Figure 3C).

### Comparison with the cancer immunome database

To better understand the 671 unique DIRAGs identified in our microarray study (Table S1) in alignment with known tumor antigens, we compared our data with the Cancer Immunome Database. Among the 1545 known antigens from the SEREX database (http://ludwig-sun5.unil.ch/CancerImmunomeDB/), 568 antigens were included in the UniPEx library. Aligning the 671 unique DIRAGs obtained in this study with these 568 SEREX antigens revealed that 103 antigens were found to overlap between lists (Table S4). Furthermore, we found that the overlap between these two lists of antigens is significant (*P* = 8.5 × 10^−8^; two-tailed Fisher's exact test).

### Comparison of DIRAGs, published CRC-specific TAAs and CRC genes

To examine whether the DIRAGs obtained (Table S1) are possibly known TAAs, we collected information about the acknowledged CRC TAAs from 8 significant articles published between 2002 and 2012 [5], [31], [32], [41], [42], [43], [44], [45], and compiled a list of 131 CRC TAAs (Table S5). Comparing the resulting list with the list of DIRAGs, we found an overlap of 7 antigens between the published CRC TAAs and the DIRAGs (Table 3). Among them, 6 antigens were found to have an increased reactivity.

**Table 3:** **List of CRC DIRAGs overlapping with published TAAs**

**DIRAG**	**Fold change**	**Upregulation/downregulation**	**Refs.**
HDAC1	1.36	↑	[32]
HIP1R	1.66	↑	[43]
HMGN2	1.71	↑	[44]
ITFG3	1.32	↑	[31]
LMNA	1.66	↑	[43]
SEC16A	0.54	↓	[31]
p53	1.75	↑	[31], [41], [43], [44], [46]

*Note*: CRC DIRAGs are found to overlap with the published TAAs. The upward and downward arrows indicate that expression of the DIRAG was found up-regulated and down-regulated, respectively, in this study. TAA, tumor-associated antigen.

In addition, a comparison between the DIRAGs (Table S1) with the CRC defined gene list (Table S6, 48 genes) showed that 7 known CRC genes were part of the list, namely *APC*, *ARID1A*, *AXIN1*, *E2F4*, *MSH2*, *PMS2*, and *TP53*. The tumor-suppressor gene *APC* is also associated with *AXIN1* in the WNT signaling pathway, which is a crucial colorectal tumorigenesis signal transduction pathway [46]. Mutations observed in *ARID1A* have been found in many tumor types including CRC [47]. Mutations at the germline DNA mismatch repair (MMR) genes like *MSH2* and *PMS2* cause hereditary non-polyposis CRC [46], whereas *TP53* somatic mutations are found in more than half of CRC cases [48].

## Discussion

Information obtained from the colon cancer immunome is of great significance, as the immune system plays a crucial role in cancer advancement [49]. Obtaining information on the molecular mechanisms in which the TAAs are involved is of great aid in understanding the biology and the mechanisms underlying the development of cancer. Furthermore, the changes in immunoreactivity or antibody-profiles provide disease-specific molecular signatures, which could be used for diagnostics and probably have additional significance to the clinical parameters currently in use for disease management. For autoantibody profiling, high-density protein arrays are a good tool for discovery, enabling a high-throughput test of many samples especially when using customized microarrays presenting selected proteins. Moreover, we have previously demonstrated (and recently Negm and colleagues used a very similar approach [50]) that purified IgG optimally conserves DIRAG profiles, thus circumventing matrix artifacts found in serum or plasma samples [36], [37].

Further bioinformatics analysis reveals that the EIF2 signaling pathway was the most overrepresented canonical pathway, which could be explained by the fact that this pathway is required to initiate protein synthesis. In addition, the EIF2 signaling pathway can also induce PI3K; in agreement, PI3K was found to be overrepresented in our study as well (Table S2).

Amplification of PI3K plays a role in the transduction of signals from extracellular stimuli, such as hormones, mitogens, growth factors and cytokines, to cellular pathways controlling cell growth, proliferation, and survival [51]. PI3K is well known to promote tumorigenesis in a variety of experimental models of cancer [49] including CRC [52]. One of the pathways activated by the amplification of PI3K is the mTOR pathway, which was also found in the top 5 canonical pathways, with 23 molecules represented in the pathway (Table 1). It is known that the mTOR pathway is activated during various cellular processes such as tumor formation and is deregulated in cancer [53]. Our results are in line with whole-exome sequencing and integrative data from TCGA network. Through analyzing the mRNA expression changes from 195 tumor samples, it was demonstrated that the PI3K, p53, and WNT pathways are deregulated in CRC [54].

One key finding of the colon cancer immunome is that factors involved in protein synthesis are enriched and overexpressed, which is confirmed by analyses performed using both IPA and WebGestalt (Hierarchical Protein Interaction Module Enrichment Analysis). Functional analyses also showed overexpression of ribosomal proteins and translation initiation factor proteins involved in the EIF2 signaling pathway (Figure 2). Furthermore, CRC DIRAGs identified were found to be enriched in proteins involved in binding functions, such as protein, ion, nucleic acid and nucleotide acid binding (Figure 3). These results are in accordance with results from Yu and colleagues [55]. In their study using CRC and adjacent normal tissues, they employed gene expression microarray analysis and also found that metabolic processes are the most common biological processes from the differential proteins analyzed (Figure 3B) [55]. As mentioned before, our results suggest that complex cellular structures are a frequent target of autoantibodies. This is further supported by the finding of enriched proteins that are known to be implicated in protein binding [9], [56], folding [57], and cell proliferation [58].

Differentially-reactive antibodies are reporters of the immune system targeting cellular as well as secreted proteins from tumors. Our results are corroborated by results obtained from Emmink and collaborators [56], who found that both extensive survival and anti-oxidant networks are represented in the secretome of colon cancer stem cells. Consistent with our findings (Figure 2), they found several ribosomal proteins and translation initiation factors and, most significantly, enriched proteins governing cell death. As a consequence, the immune response as seen in changed antibody profiles might also be driven by secreted proteins from tumors [5], as our DIRAGs are in concordance with the proteins identified in Emmink’s study [56].

In line with existing knowledge, we also found a highly significant overlap between DIRAGs (671) and the SEREX-derived antigens listed in the Cancer Immunome Database, of which 568 antigens were also present in our protein array (Fisher’s exact test: *P* = 8.5 × 10^−8^). This result demonstrates the reliability of antigenic proteins defined by our protein array. The identified antigenic proteins are mainly associated with the cell cycle, connective tissue development, transcription factors, and cell-to-cell signaling interaction networks (Table 2). It is well known that tumors reside in a microenvironment that is associated with aberrantly-altered cancer-associated cells, inflammation, hypoxia, and loss of normal tissue architecture [49], [59], further supporting our findings. The results further advocate that our approach toward identifying and characterizing antibody profiles has the potential to identify biomarkers displaying the complexity of such antigenic responses.

Screening plasma samples using our protein microarrays leads to the identification of both known (Table 3) and new TAAs, which may serve as new biomarkers. For instance, HDAC1, which plays a role in cell proliferation, survival, and inhibition of differentiation, shows higher antigenic reactivity in our study, which has been corroborated in CRC tumor studies [60], [61]. Besides, 7 known CRC genes were found by comparing the DIRAGs (Table S1) with the literature-defined 48 CRC genes (Table S6). The tumor suppressor *APC*, listed on top, interacts with *AXIN1*, which, in addition, interacts with other Wnt/ß-catenin signaling pathway components [62] and is essential for degradation of ß-catenin in the Wnt/ß-catenin signaling cascade, an important signal transduction pathway in CRC [63]. Moreover, E2F4 is an important transcription factor in cell cycle control [64], while *MSH2*, an MMR gene like *ARID1* and *PMS2* [46], [65], is highly associated with hereditary non-polyposis CRC. The MMR system recognizes and repairs mismatches between base pairs during DNA replication. *PMS2* has been found to interact with p53 [66], a transcription factor that activates apoptotic, autophagial, cell cycle arresting and cellular metabolism genes, which confers its tumor suppressor activity [46].

Taken together, our data provide a comprehensive view on the colon cancer immunome as an additional pathological layer worth considering in more detail when both bioinformatics analyses such as IPA and WebGestalt have provided overlapping information as complementary evidence. Moreover, analysis of tumor-associated antigenic proteins found in the Cancer Immunome Database provides insights into associations with cancer antigens, as well as the differentially reactive activity of antigens that are known in CRC. Further experiments to address to what extent the mechanisms involved in the antigenicity of autoantigens operate within malignancies need to be performed to deepen our understanding of interactions and networks in cancer formation. Although the antigenicity of autoantibodies to TAAs has been acknowledged in various elements of cancer growth [8], [9], additional understanding can be gathered with the aid of network and functional analyses as exemplified herein.

## Materials and methods

### Clinical information and samples

All plasma samples were collected in the ongoing molecular epidemiology “Colorectal Cancer Study of Austria” (CORSA). Since May 2002, 11,657 individuals have participated in CORSA (01/2014). The screening program “Burgenland Prevention Trial of Colorectal Disease with Immunological Testing” (B-PREDICT), which is a province-wide program, invites the public to participate in fecal occult blood testing (FOBT) annually. This invitation is open to all inhabitants of the Austrian province Burgenland, as long as they are between 40 and 80 years old. FOBT-positive individuals are offered a complete colonoscopy and, at the time of colonoscopy, are asked to take part in CORSA. A blood sample from the participants is collected as well as information in a short questionnaire. The questionnaire includes information regarding anthropometric and demographic factors, smoking status, alcohol consumption, and basic dietary habits. After sample acquisition, the heparinized plasma was centrifuged at 2000*g* for10 min, and the resulting supernatant was stored as plasma samples at ‐ 80 °C until further use.

Clinical data of CORSA participants were processed in a central database following regulated documentation guidelines. All subjects provided written informed consent. The institutional local ethics review board “Ethikkommission Burgenland” authorized the study. Further information of the study cohort is described previously [67], [68]. According to histopathology, individuals were classified as CRC cases (*n* = 32) and controls (*n* = 32). All individuals with serrated adenomas have been excluded. Controls underwent a complete colonoscopy and were found to be free of CRC and free of polyps. Persons with severe medical conditions including any other malignant condition at the initial study point were disqualified from the study (Table 4).

**Table 4:** **Demographics of the study population**

**Variable**	**CRC (n = 32)**	**Control (n = 32)**
Age	65.9 (48–82)	63.7 (40–78)
Sex		
Male	18	18
Female	14	14

Meat consumption		
Very frequent	6	5
Frequent	11	17
Seldom	13	8
None	2	2

Smoking		
Current	3	5
Former	10	8
Never	17	17
No information	2	2

Clinical tumor stage		
0	1	NA
I	8	NA
II	8	NA
III	5	NA
IV	3	NA
Missing	7	NA
Lymph node metastasis	6	NA

*Note*: Age (years) refers to the age of patients at the time of CRC diagnosis or the age of controls at the time of being recruited to the study, indicated as mean (range).

### IgG purification of blood samples

IgG purification was performed as previously described [36]. Briefly, the Melon™ Gel IgG Purification Spin Plate Kit (Thermo Scientific, Waltham, MA) was used to purify all samples according to the manufacturer’s instructions using 30 µl of plasma, followed by determination of IgG concentration as previously described [37]. Sample integrity was determined by running each purified sample on a NuPAGE® Novex 4%–12% Bis-Tris Precast Gel (Life Technologies, Carlsbad, CA).

### Protein microarray production and processing

Protein expression, purification, and microarray production were performed as previously described [36]. Briefly, the UniPEx – human in-frame cDNA protein expression library consisting of 15,417 *E. coli* cDNA expression clones and presenting 6369 unique, distinct human proteins was purchased from Imagenes (Berlin, Germany). *E. coli* culture as well as induction of protein expression were conducted in 96-well plates with minor modifications as previously described [69]. Protein expression was induced by cultivation in autoinduction medium or by adding isopropyl β-D-1-thiogalactopyranoside (IPTG). After cultivation, purification and elution of the His-tagged recombinant proteins was performed. Protein estimation was performed by running a gel [70]. Clarified *E. coli* lysates and plain buffer were used as positive and negative controls, respectively. ARChip Epoxy glass slides [71] were used to spot the protein arrays in duplicate using an Omnigrid arrayer. An illustration of the protein microarray design can be found in [70].

Protein microarray processing was done as previously described [36]. Due to array processing handling capacity, arrays were processed in 4 runs on different days with a balanced design (Day 1: 17 CRC *vs.* 17 controls; Day 2: 15 CRC *vs.* 15 controls; Day 3: 9 CRC *vs.* 9 controls; and Day 4: 9 CRC *vs.* 8 controls). A total of 50 arrays (including replicate analyses) were tested for the 32 CRC samples, and a total of 49 arrays (including replicate analyses) were tested for the 32 control samples.

### Technical performance of protein microarray analysis

We have conducted a technical study to confirm the reliability of the findings on protein microarrays by cross-wise serial mixing of 2 individuals’ IgG and then testing for significant DIRAGs of 4-fold replicates from the “pure samples at 100%” (Figure S1A). The samples from the 2 individuals show 4638 significant DIRAGS (*P* < 0.05) as illustrated by the volcano plot (Figure S1B). Correlation analysis of these 4638 significant DIRAGS with the IgG-relative amount of both individuals using the mixing-series showed that 97% DIRAGs have Pearson’s correlations >0.5 (corr = 0.5 to 1.0 or −0.5 to −1.0) and that 72% are found with a corr = 0.812 to 1.00 or −0.812 to −1.00 (histogram in Figure S1C).

### Data acquisition and statistical analysis

Array imaging, scanning, feature aligning, and gridding of spotted proteins were performed as described earlier [36] using the GenePix Pro 6.0 (Molecular Devices, Sunnyvale, CA). Briefly, correction for the systematic bias that may have been introduced using different batches of arrays was performed prior to arrangement of the protein microarray data and statistical analysis using “Distance Weighted Discrimination/DWD” as described [72]. Statistical analysis of the microarray data was carried out using R 3.0.1 and BRB-ArrayTools 4.3.1 [73]. Sample size calculation was conducted using the BRB-ArrayTools plug-in with a significance level of 0.001 (*α*), a power of 0.75 (1 − *β*), and a fold change of 2. An expected sample size in each class of 32 was determined by applying the 50th percentile of the variance distribution. DIRAGs between the patients and controls were defined using the Class Comparison tool in BRB-ArrayTools with *P <* 0.01. A file was prepared for the resulting analysis data, which included both a list of DIRAGs ID annotations and its ratio of the geometric means between sample groups (Table S1).

### Pathway analysis

IPA was used for the generation of “Core Analyses” to interpret the data in relation to biological networks, biological processes, and pathways using the Ingenuity Knowledge Base reference set. The analyzed canonical pathways were ordered by the ratio (features in a given pathway meeting the selection criteria, divided by the total number of features included in that pathway) and the Fisher’s Exact test *P* value.

### Web-based gene set analysis toolkit

The Web-based GEne SeT AnaLysis Toolkit (WebGestalt; http://bioinfo.vanderbilt.edu/webgestalt/) [74], [75] was used for hierarchical enrichment analyses of protein interaction networks and Gene Ontology (GO) slim classification for creating bar charts with respect to biological processes, molecular functions, and cellular components. The hypergeometric test was used for enrichment analysis, and adjustment for multiple testing was achieved using the Benjamini & Hochberg procedure. The significance level was adjusted to the top 10 pathways (*P* < 0.05), and a minimal base amount of two genes for a category was set.

### Cancer immunome database comparison

Comparisons with the entire 1545 SEREX antigens enlisted in the Cancer Immunome Database were performed (http://ludwig-sun5.unil.ch/CancerImmunomeDB/). The complete unique list of SEREX antigens was matched to the DIRAGs table (Table S1), searching for already known antigens. Fisher’s exact test (two-tailed) was used to test for significant enrichment of antigens found in our study and the SEREX antigens present in the UniPEx library. Statistical tests were done using RStudio software (version 0.97.551).

### TAA literature review and comparison with CRC genes

To identify DIRAGs from this study that are possibly acknowledged as TAAs, a table of the known CRC TAAs was compiled from several publications [5], [12], [24], [41], [42], [43], [44], [45], [54]. This list (Table S5) was intersected with the DIRAGs (Table S1). Additionally, a compilation of the most recognized CRC genes listed in the literature was generated from reviews [46], [48], [76]. To further complement this list, we added information of the cancer census gene mutation data from the Catalogue Of Somatic Mutations In Cancer (COSMIC) website, http://www.sanger.ac.uk/cosmic [77]. Only somatic gene mutations detected in CRC were considered. With the found CRC genes, a table was generated (Table S6) and the overlapping antigens with those identified in our experiments were examined.

## Authors’ contributions

JL performed the study, analyzed and interpreted the data, and wrote the manuscript. JL, KS and PeH processed the protein microarrays and performed data analysis. IG interpreted data and helped with the manuscript writing. AG, PhH, and SB conducted biobanking, sample, and data management, and helped with study design. GL and KM coordinated patient recruitment and clinical examination of patients. AW designed the study, coordinated, interpreted, supervised and corrected the manuscript. All authors read and approved the final manuscript.

## Competing interest

The authors have declared no competing interests.

## Supplementary material

Supplementary Figure S1 **Reliability analysis of protein microarraysA.** Purified IgG from 2 different randomly-selected control samples was mixed together in various percentages with 3 or 4 replicates for each combination. **B.** These resulting mixtures were then analyzed for their abilities to evaluate 4638 DIRAGs between these 2 samples. **C.** Pearson correlation with respect to the relative content of each sample in the mixture (%) was calculated. We found that 71.6% DIRAGs (3324) have an absolute correlation coefficient ranging from 0.812 to 1.00 or −0.812 to −1.00, while 4499 DIRAGs (97%) have an absolute correlation coefficient ranging from 0.5 to 1.0 or −0.5 to −1.0.

Supplementary Figure S2 **Directed acyclic graph of the DIRAGs**Visualization of the hierarchical relationship of enriched phenotypes terms with WebGestalt. The enriched network modules are indicated in red, the most representative modules are highlighted in dark red (Modules 1, 2, and 3) while their non-enriched parents are shown in black. Enrichment analysis was performed using the hypergeometric test, and the Benjamini–Hochberg procedure was used for multiple test adjustment. A minimum number of two genes for a category was used as cut-off, and pathways significance level was set at *P* = 0.01 (*t*-test).

Supplementary Table S1 **List of DIRAGs obtained from the class comparison between CRC and control samples (*P* = 0.01)**

Supplementary Table S2 **Complete list of 50 pathways enriched with DIRAGs analyzed using IPA (*P* < 0.01)**

Supplementary Table S3 **Top 5 network functions and the associated DIRAGs analyzed using IPA**

Supplementary Table S4 **List of antigens overlapping between DIRAGs identified in this study and SEREX antigens**

Supplementary Table S5 **Compilation of CRC TAAs from literature**

Supplementary Table S6 **List of CRC-associated genes**
