# Supplementary material for: The Immunome of Colon Cancer: Functional In Silico Analysis of Antigenic Proteins Deduced from IgG Microarray Profiling
Source: Genomics Proteomics Bioinformatics. 2018 Mar 2;16(1):73–84. doi: 10.1016/j.gpb.2017.10.002 (PMC6000238; doi:10.1016/j.gpb.2017.10.002)
Supplement: Supplementary Figure S1 — Reliability analysis of protein microarraysA. Purified IgG from 2 different randomly-selected control samples was mixed together in various percentages with 3 or 4 replicates for each combination. B. These resulting mixtures were then analyzed for their abilities to evaluate 4638 DIRAGs between these 2 samples. C. Pearson correlation with respect to the relative content of each sample in the mixture (%) was calculated. We found that 71.6% DIRAGs (3324) have an absolute correlation coefficient ranging from 0.812 to 1.00 or −0.812 to −1.00, while 4499 DIRAGs (97%) have an absolute correlation coefficient ranging from 0.5 to 1.0 or −0.5 to −1.0. [file mmc1.pptx]

## Slide 1
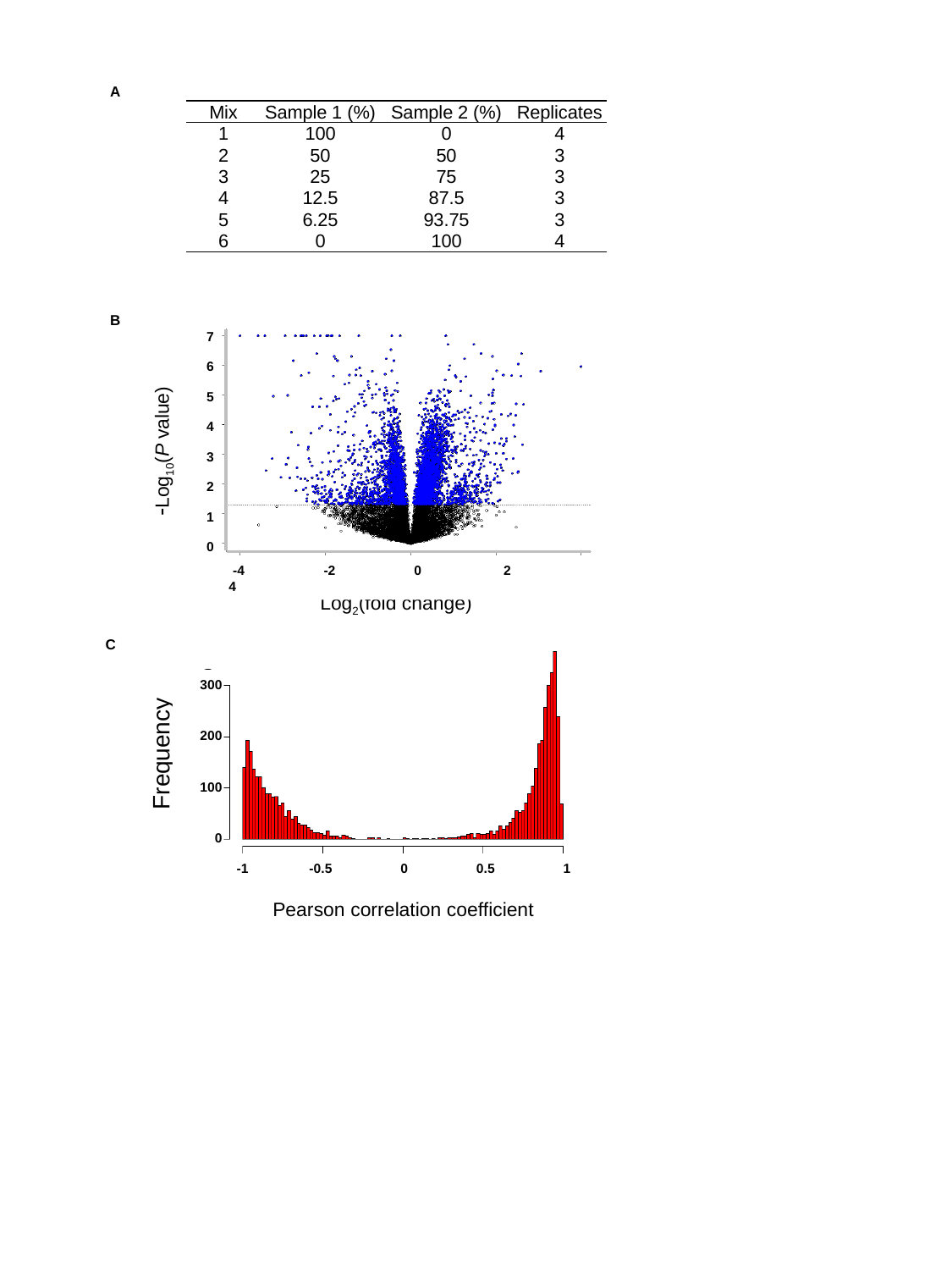

A
| Mix | Sample 1 (%) | Sample 2 (%) | Replicates |
| --- | --- | --- | --- |
| 1 | 100 | 0 | 4 |
| 2 | 50 | 50 | 3 |
| 3 | 25 | 75 | 3 |
| 4 | 12.5 | 87.5 | 3 |
| 5 | 6.25 | 93.75 | 3 |
| 6 | 0 | 100 | 4 |
 B
7
6
5
4
3
2
1
0
-Log10(P value)
 -4 -2 0 2 4
Log2(fold change)
 C
300
200
100
0
Frequency
0 100 200 300
 -1 -0.5 0 0.5 1
Pearson correlation coefficient
