# Supplementary figures and images for: The Immunome of Colon Cancer: Functional In Silico Analysis of Antigenic Proteins Deduced from IgG Microarray Profiling
Source: Genomics Proteomics Bioinformatics. 2018 Mar 2;16(1):73–84. doi: 10.1016/j.gpb.2017.10.002 (PMC6000238; doi:10.1016/j.gpb.2017.10.002)

## Slide 1
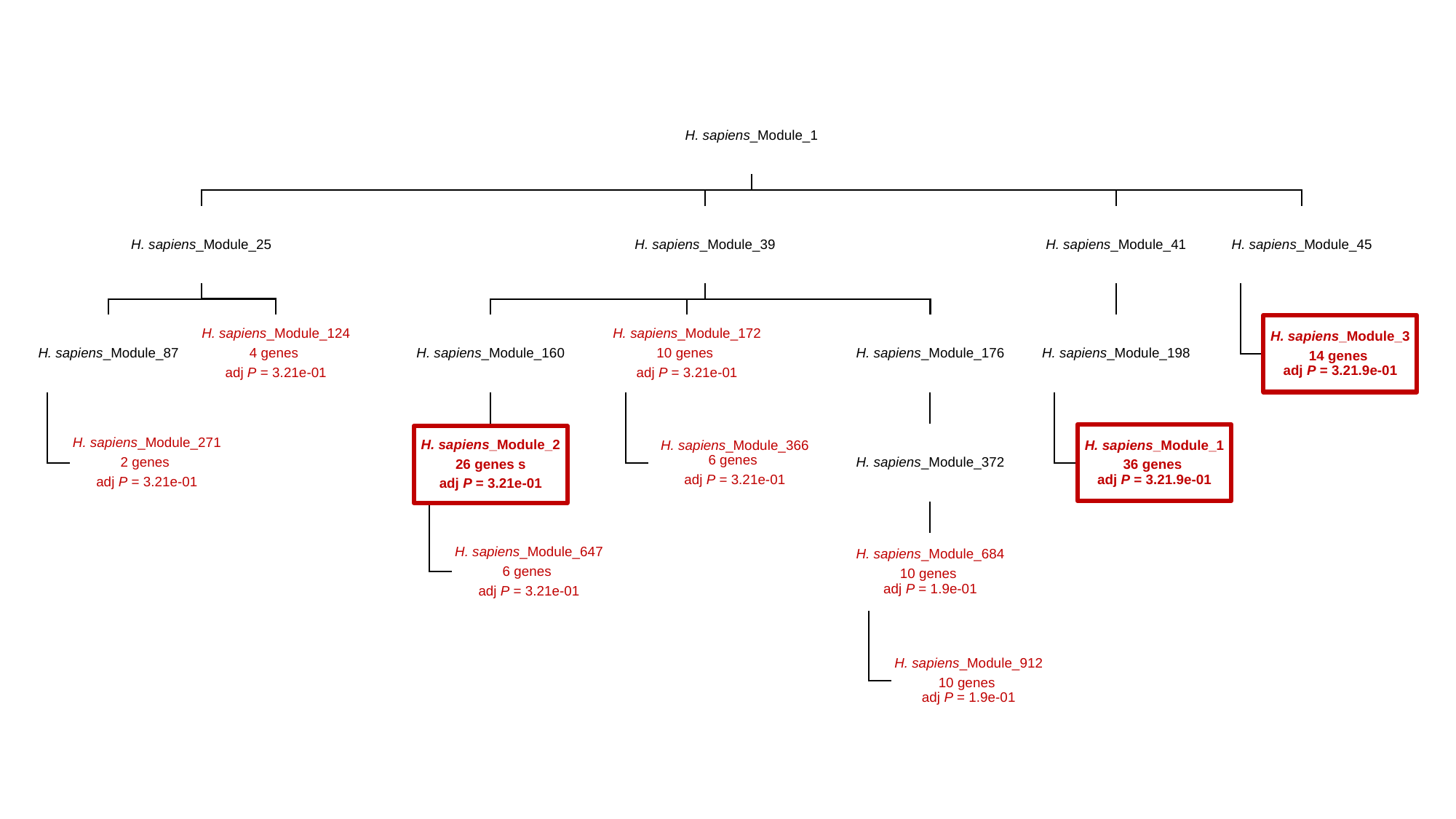

Supplement: Supplementary Figure S2 — Directed acyclic graph of the DIRAGsVisualization of the hierarchical relationship of enriched phenotypes terms with WebGestalt. The enriched network modules are indicated in red, the most representative modules are highlighted in dark red (Modules 1, 2, and 3) while their non-enriched parents are shown in black. Enrichment analysis was performed using the hypergeometric test, and the Benjamini–Hochberg procedure was used for multiple test adjustment. A minimum number of two genes for a category was used as cut-off, and pathways significance level was set at P = 0.01 (t-test). [file mmc2.pptx]
