# Supplementary material for: The Immunome of Colon Cancer: Functional In Silico Analysis of Antigenic Proteins Deduced from IgG Microarray Profiling
Source: Genomics Proteomics Bioinformatics. 2018 Mar 2;16(1):73–84. doi: 10.1016/j.gpb.2017.10.002 (PMC6000238; doi:10.1016/j.gpb.2017.10.002)
Supplement: Supplementary Table S1 — List of DIRAGs obtained from the class comparison between CRC and control samples (P = 0.01) [file mmc3.docx]

**Table S1 List of DIRAGs obtained from the class comparison between CRC and control samples (*P* = 0.01)**

| **DIRAG** | **Parametric *P* value** | **Fold change** | **Upregulation/downregulation** |
| --- | --- | --- | --- |
| ABCA3 | 0.0049215 | 1.5 | 🡹 |
| ACAA2 | 0.0003410 | 1.96 | 🡹 |
| ACAD9 | 0.0004178 | 1.58 | 🡹 |
| ACADVL | 0.0074404 | 1.46 | 🡹 |
| ACAP1 | 0.0092975 | 1.46 | 🡹 |
| ACIN1 | 0.0010577 | 1.44 | 🡹 |
| ACSS1 | 0.0099031 | 1.58 | 🡹 |
| ACTL6B | 0.0073551 | 0.68 | 🡻 |
| ADAM15 | 0.0015854 | 1.53 | 🡹 |
| ADAM8 | 0.0027221 | 1.39 | 🡹 |
| ADAR | 0.0026671 | 1.26 | 🡹 |
| ADD2 | 0.0020015 | 1.66 | 🡹 |
| AES | 0.0093765 | 1.3 | 🡹 |
| AHCTF1 | 0.0047618 | 1.6 | 🡹 |
| AHCTF1 | 0.0090241 | 1.54 | 🡹 |
| AHNAK | 0.0087626 | 1.49 | 🡹 |
| AIDA | 0.0083525 | 1.31 | 🡹 |
| AIP | 0.0005183 | 1.3 | 🡹 |
| AKR1C4 | 0.0020241 | 1.72 | 🡹 |
| ALDOC | 0.0023928 | 1.29 | 🡹 |
| ANAPC2 | 0.0056709 | 1.35 | 🡹 |
| ANGPTL2 | 0.0000144 | 1.6 | 🡹 |
| ANKRD11 | 0.0018714 | 1.33 | 🡹 |
| ANKRD11 | 0.0022362 | 1.37 | 🡹 |
| ANKRD12 | 0.0036532 | 1.55 | 🡹 |
| ANKRD52 | 0.0041759 | 1.45 | 🡹 |
| ANKZF1 | 0.0016366 | 1.54 | 🡹 |
| ANO9 | 0.0040514 | 1.64 | 🡹 |
| ANXA7 | 0.0094038 | 1.38 | 🡹 |
| AP1G1 | 0.0067068 | 1.4 | 🡹 |
| AP1G2 | 0.0054444 | 1.59 | 🡹 |
| AP3D1 | 0.0061748 | 1.24 | 🡹 |
| APBA3 | 0.0078912 | 1.29 | 🡹 |
| APC | 0.0059049 | 1.42 | 🡹 |
| ARCN1 | 0.0015899 | 1.4 | 🡹 |
| ARFIP2 | 0.0024590 | 1.39 | 🡹 |
| ARHGAP21 | 0.0097578 | 1.5 | 🡹 |
| ARHGAP26 | 0.0059011 | 1.38 | 🡹 |
| ARHGEF1 | 0.0008732 | 1.47 | 🡹 |
| ARHGEF1 | 0.0060010 | 1.62 | 🡹 |
| ARHGEF16 | 0.0019782 | 1.53 | 🡹 |
| ARHGEF3 | 0.0012127 | 1.39 | 🡹 |
| ARHGEF9 | 0.0004967 | 1.63 | 🡹 |
| ARID1A | 0.0027783 | 1.35 | 🡹 |
| ARID1A | 0.0081430 | 1.32 | 🡹 |
| ARID1B | 0.0083295 | 1.4 | 🡹 |
| ARID5A | 0.0086424 | 1.22 | 🡹 |
| ARPC1B | 0.0033769 | 1.4 | 🡹 |
| ARRB2 | 0.0067769 | 1.38 | 🡹 |
| ASPSCR1 | 0.0051838 | 1.35 | 🡹 |
| ATAD3A | 0.0067263 | 1.35 | 🡹 |
| ATP11B | 0.0012529 | 1.48 | 🡹 |
| ATP5G3 | 0.0057932 | 1.37 | 🡹 |
| ATP5H | 0.0002271 | 1.51 | 🡹 |
| ATXN1 | 0.0059098 | 1.6 | 🡹 |
| ATXN2L | 0.0007014 | 1.56 | 🡹 |
| AURKAIP1 | 0.0059343 | 1.3 | 🡹 |
| AXIN1 | 0.0064303 | 1.42 | 🡹 |
| AZI1 | 0.0055988 | 1.37 | 🡹 |
| BABAM1 | 0.0068899 | 0.49 | 🡻 |
| BAG1 | 0.0013517 | 1.43 | 🡹 |
| BAG1 | 0.0031010 | 0.5 | 🡻 |
| BAG6 | 0.0063961 | 1.29 | 🡹 |
| BAZ1A | 0.0001398 | 1.39 | 🡹 |
| BCAS2 | 0.0000373 | 1.5 | 🡹 |
| BCL9 | 0.0065593 | 1.42 | 🡹 |
| BCLAF1 | 0.0000112 | 1.63 | 🡹 |
| BCLAF1 | 0.0001962 | 1.29 | 🡹 |
| BCR | 0.0013968 | 1.36 | 🡹 |
| BHLHE40 | 0.0059081 | 1.45 | 🡹 |
| BIN3 | 0.0010048 | 1.53 | 🡹 |
| BIN3 | 0.0062335 | 1.5 | 🡹 |
| BLMH | 0.0093863 | 1.23 | 🡹 |
| BRD3 | 0.0016797 | 1.34 | 🡹 |
| BRPF1 | 0.0015174 | 1.36 | 🡹 |
| BRPF1 | 0.0052106 | 1.53 | 🡹 |
| C10orf2 | 0.0077413 | 0.65 | 🡻 |
| C11orf30 | 0.0014153 | 1.44 | 🡹 |
| C12orf35 | 0.0083244 | 1.44 | 🡹 |
| C14orf38 | 0.0035456 | 1.4 | 🡹 |
| C16orf58 | 0.0050675 | 1.51 | 🡹 |
| C17orf101 | 0.0049733 | 1.48 | 🡹 |
| C17orf85 | 0.0006825 | 1.71 | 🡹 |
| C18orf21 | 0.0041968 | 1.32 | 🡹 |
| C19orf21 | 0.0000691 | 1.96 | 🡹 |
| C8orf37 | 0.0048897 | 1.39 | 🡹 |
| C9orf86 | 0.0037912 | 1.54 | 🡹 |
| CABIN1 | 0.0062582 | 1.47 | 🡹 |
| CAP1 | 0.0014670 | 1.39 | 🡹 |
| CAPG | 0.0005078 | 1.48 | 🡹 |
| CAPN2 | 0.0073887 | 1.38 | 🡹 |
| CAPRIN1 | 0.0011081 | 1.65 | 🡹 |
| CAPRIN1 | 0.0093224 | 1.48 | 🡹 |
| CARD8 | 0.0075546 | 1.5 | 🡹 |
| CASP1 | 0.0088782 | 1.5 | 🡹 |
| CASP8 | 0.0010950 | 1.52 | 🡹 |
| CASP8 | 0.0023364 | 1.54 | 🡹 |
| CCDC115 | 0.0018310 | 1.31 | 🡹 |
| CCDC64 | 0.0000833 | 1.9 | 🡹 |
| CCDC64 | 0.0016014 | 1.38 | 🡹 |
| CCDC88C | 0.0055865 | 1.5 | 🡹 |
| CCL28 | 0.0001699 | 2.13 | 🡹 |
| CCND1 | 0.0000382 | 1.97 | 🡹 |
| CCNL1 | 0.0002634 | 1.54 | 🡹 |
| CCT5 | 0.0090872 | 1.38 | 🡹 |
| CCT6A | 0.0000401 | 1.67 | 🡹 |
| CD97 | 0.0042362 | 1.64 | 🡹 |
| CDH2 | 0.0016751 | 1.88 | 🡹 |
| CDK16 | 0.0000455 | 1.49 | 🡹 |
| CDK16 | 0.0008126 | 1.53 | 🡹 |
| CDK5RAP1 | 0.0025008 | 1.48 | 🡹 |
| CDK9 | 0.0019008 | 1.53 | 🡹 |
| CDR2 | 0.0029744 | 1.56 | 🡹 |
| CEP164 | 0.0081379 | 1.58 | 🡹 |
| CHD3 | 0.0044903 | 1.31 | 🡹 |
| CHMP1B | 0.0005329 | 1.72 | 🡹 |
| CIAO1 | 0.0019682 | 1.77 | 🡹 |
| CIC | 0.0076877 | 1.48 | 🡹 |
| CKMT1A | 0.0041705 | 1.36 | 🡹 |
| CLCN3 | 0.0099382 | 1.3 | 🡹 |
| CLIP1 | 0.0051250 | 1.45 | 🡹 |
| CLIP2 | 0.0043215 | 1.47 | 🡹 |
| CLSTN3 | 0.0012385 | 1.34 | 🡹 |
| CLTA | 0.0049419 | 1.52 | 🡹 |
| CLU | 0.0025490 | 1.4 | 🡹 |
| CLU | 0.0092867 | 1.38 | 🡹 |
| CMTM3 | 0.0008224 | 1.61 | 🡹 |
| CNOT2 | 0.0050783 | 1.55 | 🡹 |
| COL1A2 | 0.0098304 | 1.5 | 🡹 |
| COL3A1 | 0.0028810 | 1.47 | 🡹 |
| COL3A1 | 0.0044698 | 1.36 | 🡹 |
| COL6A1 | 0.0093175 | 1.53 | 🡹 |
| COQ6 | 0.0038154 | 1.48 | 🡹 |
| CORO7 | 0.0039374 | 1.48 | 🡹 |
| COX6B1 | 0.0056288 | 1.47 | 🡹 |
| COX7A2L | 0.0000721 | 1.55 | 🡹 |
| CPE | 0.0083883 | 1.47 | 🡹 |
| CPLX1 | 0.0036850 | 1.33 | 🡹 |
| CPNE1 | 0.0005228 | 1.48 | 🡹 |
| CPNE1 | 0.0060175 | 1.23 | 🡹 |
| CPNE8 | 0.0026788 | 1.73 | 🡹 |
| CRNKL1 | 0.0057307 | 1.37 | 🡹 |
| CSF1R | 0.0005444 | 1.46 | 🡹 |
| CTAGE5 | 0.0027729 | 1.52 | 🡹 |
| CTDP1 | 0.0094092 | 1.46 | 🡹 |
| CTIF | 0.0017318 | 1.55 | 🡹 |
| CTNNA1 | 0.0017328 | 1.39 | 🡹 |
| CTNNA1 | 0.0098252 | 1.42 | 🡹 |
| CTNND1 | 0.0091573 | 1.31 | 🡹 |
| CUL1 | 0.0006975 | 1.73 | 🡹 |
| CYB5B | 0.0017445 | 1.46 | 🡹 |
| CYB5B | 0.0052952 | 1.24 | 🡹 |
| CYB5R3 | 0.0083622 | 1.43 | 🡹 |
| CYC1 | 0.0038140 | 1.63 | 🡹 |
| CYHR1 | 0.0056041 | 1.5 | 🡹 |
| DAXX | 0.0024465 | 1.27 | 🡹 |
| DBNL | 0.0004704 | 1.9 | 🡹 |
| DBNL | 0.0020252 | 1.41 | 🡹 |
| DCAF5 | 0.0080103 | 1.27 | 🡹 |
| DCAF5 | 0.0097296 | 1.21 | 🡹 |
| DCHS1 | 0.0057712 | 1.39 | 🡹 |
| DCTPP1 | < 1e-07 | 2.73 | 🡹 |
| DDIT4 | 0.0059757 | 1.41 | 🡹 |
| DDX1 | 0.0021731 | 1.4 | 🡹 |
| DDX21 | 0.0056734 | 1.39 | 🡹 |
| DDX23 | 0.0022255 | 1.59 | 🡹 |
| DDX24 | 0.0002165 | 1.74 | 🡹 |
| DDX27 | 0.0052263 | 1.44 | 🡹 |
| DEK | 0.0024177 | 0.43 | 🡻 |
| DFFA | 0.0004365 | 1.37 | 🡹 |
| DGKZ | 0.0001205 | 1.84 | 🡹 |
| DHX9 | 0.0001036 | 1.42 | 🡹 |
| DHX9 | 0.0052535 | 1.47 | 🡹 |
| DIAPH1 | 0.0000972 | 1.84 | 🡹 |
| DIDO1 | 0.0002275 | 1.84 | 🡹 |
| DIS3L | 0.0055593 | 1.42 | 🡹 |
| DLD | 0.0026456 | 1.26 | 🡹 |
| DNAJA1 | 0.0019309 | 1.42 | 🡹 |
| DNAJA2 | 0.0022608 | 1.5 | 🡹 |
| DNAJC10 | 0.0024887 | 1.43 | 🡹 |
| DOCK9 | 0.0020494 | 0.59 | 🡻 |
| DPF2 | 0.0025790 | 1.87 | 🡹 |
| DPF2 | 0.0081186 | 1.33 | 🡹 |
| DPPA4 | 0.0009039 | 0.37 | 🡻 |
| DPYSL3 | 0.0005504 | 1.55 | 🡹 |
| DUSP1 | 0.0088773 | 1.39 | 🡹 |
| DUSP10 | 0.0057309 | 1.4 | 🡹 |
| DUSP14 | 0.0071171 | 1.3 | 🡹 |
| DYNC1H1 | 0.0000051 | 1.99 | 🡹 |
| DYNLT1 | 0.0001104 | 1.58 | 🡹 |
| E2F4 | 0.0000103 | 2.12 | 🡹 |
| ECHDC1 | 0.0092312 | 1.35 | 🡹 |
| EDC4 | 0.0066014 | 0.57 | 🡻 |
| EDC4 | 0.0099081 | 1.26 | 🡹 |
| EEF1D | 0.0008078 | 1.46 | 🡹 |
| EEF1D | 0.0009472 | 1.55 | 🡹 |
| EEF1D | 0.0034052 | 1.61 | 🡹 |
| EEF1D | 0.0046032 | 1.39 | 🡹 |
| EGR1 | 0.0024778 | 1.4 | 🡹 |
| EGR1 | 0.0057888 | 1.41 | 🡹 |
| EHD1 | 0.0071728 | 1.5 | 🡹 |
| EHD4 | 0.0050128 | 1.29 | 🡹 |
| EHMT2 | 0.0054593 | 1.64 | 🡹 |
| EID1 | 0.0011879 | 1.52 | 🡹 |
| EID1 | 0.0091872 | 1.52 | 🡹 |
| EIF3F | 0.0096661 | 0.48 | 🡻 |
| EIF3G | 0.0089482 | 1.35 | 🡹 |
| EIF4G1 | 0.0028658 | 1.42 | 🡹 |
| EIF4G2 | 0.0001274 | 1.63 | 🡹 |
| ELF1 | 0.0003295 | 2.08 | 🡹 |
| ELK1 | 0.0014256 | 1.48 | 🡹 |
| ELMO2 | 0.0065972 | 1.35 | 🡹 |
| EPHB3 | 0.0063856 | 1.48 | 🡹 |
| ERBB3 | 0.0055765 | 1.55 | 🡹 |
| ERCC3 | 0.0037140 | 1.31 | 🡹 |
| ERF | 0.0008626 | 1.68 | 🡹 |
| ERP29 | 0.0087358 | 1.3 | 🡹 |
| ETS1 | 0.0000701 | 1.82 | 🡹 |
| ETS1 | 0.0006031 | 1.65 | 🡹 |
| EXOSC1 | 0.0091792 | 1.44 | 🡹 |
| EXOSC7 | 0.0000075 | 1.8 | 🡹 |
| EXOSC7 | 0.0000918 | 1.45 | 🡹 |
| EXOSC8 | 0.0010895 | 1.51 | 🡹 |
| FAM100B | 0.0050478 | 1.51 | 🡹 |
| FAM117A | 0.0008640 | 1.53 | 🡹 |
| FAM129A | 0.0028950 | 1.4 | 🡹 |
| FAM134A | 0.0056093 | 1.34 | 🡹 |
| FAM13A | 0.0000227 | 1.99 | 🡹 |
| FAM160A2 | 0.0087342 | 1.5 | 🡹 |
| FAM204A | 0.0009622 | 1.56 | 🡹 |
| FAM21A | 0.0054960 | 1.28 | 🡹 |
| FAM48A | 0.0080190 | 1.69 | 🡹 |
| FAM60A | 0.0045492 | 1.29 | 🡹 |
| FAM65A | 0.0030721 | 1.55 | 🡹 |
| FAM65B | 0.0043234 | 1.38 | 🡹 |
| FAM98A | 0.0041619 | 1.36 | 🡹 |
| FBLL1 | 0.0062266 | 1.37 | 🡹 |
| FBXO41 | 0.0025371 | 1.42 | 🡹 |
| FBXO7 | 0.0089278 | 1.39 | 🡹 |
| FCGBP | 0.0066519 | 1.47 | 🡹 |
| FCHSD1 | 0.0023320 | 1.42 | 🡹 |
| FERMT3 | 0.0008821 | 1.64 | 🡹 |
| FKBP15 | 0.0008581 | 1.27 | 🡹 |
| FKBP8 | 0.0034487 | 1.37 | 🡹 |
| FLNB | 0.0000148 | 1.85 | 🡹 |
| FLOT1 | 0.0063902 | 1.41 | 🡹 |
| FNDC1 | 0.0056994 | 1.41 | 🡹 |
| FPGS | 0.0004921 | 1.55 | 🡹 |
| GAA | 0.0017257 | 1.7 | 🡹 |
| GBP2 | 0.0001659 | 1.86 | 🡹 |
| GBP5 | 0.0000001 | 2.24 | 🡹 |
| GCC2 | 0.0002192 | 2.04 | 🡹 |
| GDI1 | 0.0067392 | 1.51 | 🡹 |
| GIMAP5 | 0.0005179 | 1.85 | 🡹 |
| GLOD4 | 0.0044579 | 1.6 | 🡹 |
| GLUL | 0.0031120 | 1.38 | 🡹 |
| GNAO1 | 0.0003393 | 1.62 | 🡹 |
| GNB2 | 0.0074996 | 1.35 | 🡹 |
| GNL3 | 0.0098511 | 1.35 | 🡹 |
| GOSR1 | 0.0099640 | 1.44 | 🡹 |
| GPI | 0.0001043 | 1.46 | 🡹 |
| GPI | 0.0031429 | 1.45 | 🡹 |
| GPSM1 | 0.0008887 | 1.65 | 🡹 |
| GRAMD1A | 0.0030577 | 1.35 | 🡹 |
| GRASP | 0.0055365 | 1.45 | 🡹 |
| GSDMD | 0.0000500 | 1.81 | 🡹 |
| GSDMD | 0.0051307 | 1.45 | 🡹 |
| GSTP1 | 0.0077874 | 1.46 | 🡹 |
| GTF3C1 | 0.0002173 | 1.31 | 🡹 |
| GYS1 | 0.0020213 | 1.5 | 🡹 |
| HAPLN3 | 0.0001940 | 1.64 | 🡹 |
| HAPLN3 | 0.0022636 | 1.48 | 🡹 |
| HARS | 0.0083037 | 1.47 | 🡹 |
| HCLS1 | 0.0005803 | 1.41 | 🡹 |
| HCLS1 | 0.0036529 | 1.34 | 🡹 |
| HDAC1 | 0.0009574 | 1.36 | 🡹 |
| HEATR8 | 0.0081150 | 1.49 | 🡹 |
| HEXDC | 0.0096890 | 0.57 | 🡻 |
| HGS | 0.0003066 | 1.57 | 🡹 |
| HIC1 | 0.0027189 | 1.36 | 🡹 |
| HIP1R | 0.0017387 | 1.66 | 🡹 |
| HK1 | 0.0001920 | 2.14 | 🡹 |
| HLA-A | 0.0078822 | 1.53 | 🡹 |
| HLA-A | 0.0094216 | 1.42 | 🡹 |
| HLA-B | 0.0051523 | 1.43 | 🡹 |
| HLA-C | 0.0003984 | 1.44 | 🡹 |
| HLA-C | 0.0009147 | 1.47 | 🡹 |
| HMG20B | 0.0047486 | 1.58 | 🡹 |
| HMGN2 | 0.0023737 | 1.71 | 🡹 |
| HNRNPA2B1 | 0.0000847 | 1.7 | 🡹 |
| HNRNPA3 | 0.0061563 | 1.31 | 🡹 |
| HNRNPAB | 0.0063638 | 1.34 | 🡹 |
| HNRNPC | 0.0002540 | 1.42 | 🡹 |
| HNRNPM | 0.0079494 | 1.43 | 🡹 |
| HNRPDL | 0.0094208 | 1.31 | 🡹 |
| HSD17B4 | 0.0019946 | 1.42 | 🡹 |
| HSP90AB1 | 0.0000011 | 1.99 | 🡹 |
| HSPA1A | 0.0085320 | 1.22 | 🡹 |
| HTATIP2 | 0.0048194 | 1.38 | 🡹 |
| IDH3B | 0.0015734 | 1.43 | 🡹 |
| IDS | 0.0084318 | 0.64 | 🡻 |
| IL16 | 0.0013220 | 1.33 | 🡹 |
| IL32 | 0.0060983 | 1.42 | 🡹 |
| IL4R | 0.0056052 | 1.28 | 🡹 |
| ILF3 | 0.0074599 | 1.43 | 🡹 |
| IMP4 | 0.0000034 | 1.62 | 🡹 |
| IMPDH2 | 0.0007998 | 1.31 | 🡹 |
| ING3 | 0.0000511 | 1.83 | 🡹 |
| INTS1 | 0.0015244 | 1.44 | 🡹 |
| IQGAP1 | 0.0004554 | 1.47 | 🡹 |
| IRF8 | 0.0061459 | 1.51 | 🡹 |
| IRF9 | 0.0004924 | 1.53 | 🡹 |
| ISG15 | 0.0000016 | 2.17 | 🡹 |
| IST1 | 0.0042018 | 1.3 | 🡹 |
| ITFG3 | 0.0027583 | 1.32 | 🡹 |
| ITGB1BP1 | 0.0088655 | 1.31 | 🡹 |
| IVNS1ABP | 0.0058972 | 1.77 | 🡹 |
| JMJD7-PLA2G4B | 0.0053928 | 1.43 | 🡹 |
| JUNB | 0.0034773 | 1.42 | 🡹 |
| JUND | 0.0015396 | 1.42 | 🡹 |
| KAT6A | 0.0082857 | 1.38 | 🡹 |
| KCTD13 | 0.0002351 | 1.5 | 🡹 |
| KCTD2 | 0.0005724 | 1.49 | 🡹 |
| KCTD5 | 0.0027984 | 1.46 | 🡹 |
| KDM4A | 0.0032567 | 1.42 | 🡹 |
| KHDRBS1 | 0.0018685 | 1.64 | 🡹 |
| KIAA0020 | 0.0048968 | 1.36 | 🡹 |
| KIAA0913 | 0.0063743 | 0.57 | 🡻 |
| KIAA1430 | 0.0008827 | 1.4 | 🡹 |
| KIDINS220 | 0.0001150 | 1.72 | 🡹 |
| KIF2A | 0.0000366 | 1.98 | 🡹 |
| KLF2 | 0.0006113 | 1.86 | 🡹 |
| KLF2 | 0.0028418 | 1.57 | 🡹 |
| KLF6 | 0.0018549 | 1.61 | 🡹 |
| KPNA2 | 0.0059868 | 1.39 | 🡹 |
| KPNA2 | 0.0093912 | 0.67 | 🡻 |
| KRBA1 | 0.0003753 | 1.6 | 🡹 |
| KRT8 | 0.0097043 | 1.32 | 🡹 |
| KSR1 | 0.0010039 | 1.38 | 🡹 |
| LAMA5 | 0.0006066 | 1.65 | 🡹 |
| LAMB3 | 0.0004284 | 1.5 | 🡹 |
| LARP1 | 0.0030551 | 1.89 | 🡹 |
| LARP4 | 0.0016393 | 1.39 | 🡹 |
| LARS | 0.0050040 | 1.61 | 🡹 |
| LCK | 0.0049302 | 0.53 | 🡻 |
| LDB1 | 0.0095723 | 1.4 | 🡹 |
| LDLR | 0.0002314 | 1.31 | 🡹 |
| LGALS3 | 0.0003652 | 1.63 | 🡹 |
| LIMD2 | 0.0078051 | 0.67 | 🡻 |
| LLGL2 | 0.0018584 | 1.44 | 🡹 |
| LMNA | 0.0023700 | 1.66 | 🡹 |
| LMO3 | 0.0084575 | 1.38 | 🡹 |
| LOC100130899 | 0.0027141 | 1.32 | 🡹 |
| LOC100132116 | 0.0052327 | 1.4 | 🡹 |
| LOC100506012 | 0.0005830 | 1.46 | 🡹 |
| LOC644762 | 0.0010403 | 1.42 | 🡹 |
| LOC644961 | 0.0070379 | 1.51 | 🡹 |
| LPCAT1 | 0.0002333 | 1.82 | 🡹 |
| LPIN2 | 0.0093960 | 1.34 | 🡹 |
| LRIG1 | 0.0087000 | 1.43 | 🡹 |
| LRP1 | 0.0040797 | 1.42 | 🡹 |
| LSM14A | 0.0026988 | 1.7 | 🡹 |
| LSM14B | 0.0041704 | 1.53 | 🡹 |
| LTA4H | 0.0011759 | 1.55 | 🡹 |
| LTBP2 | 0.0048282 | 1.42 | 🡹 |
| LTBP3 | 0.0013057 | 1.49 | 🡹 |
| LUC7L3 | 0.0025998 | 1.36 | 🡹 |
| LYAR | 0.0028749 | 1.53 | 🡹 |
| LZTS2 | 0.0034449 | 1.42 | 🡹 |
| MAF1 | 0.0001332 | 1.6 | 🡹 |
| MAN2B2 | 0.0055922 | 1.34 | 🡹 |
| MANSC1 | 0.0027379 | 1.41 | 🡹 |
| MAP1S | 0.0003788 | 1.65 | 🡹 |
| MAP4 | 0.0027741 | 1.37 | 🡹 |
| MAP7D1 | 0.0056395 | 1.42 | 🡹 |
| MAPRE1 | 0.0006509 | 1.47 | 🡹 |
| MAST2 | 0.0040977 | 1.56 | 🡹 |
| MAZ | 0.0030726 | 1.76 | 🡹 |
| MAZ | 0.0077408 | 1.36 | 🡹 |
| MBD1 | 0.0011078 | 1.74 | 🡹 |
| MCM6 | 0.0099259 | 1.36 | 🡹 |
| MED21 | 0.0095891 | 1.46 | 🡹 |
| MED4 | 0.0041429 | 1.41 | 🡹 |
| MEGF8 | 0.0036779 | 1.22 | 🡹 |
| MFN2 | 0.0078628 | 1.29 | 🡹 |
| MICAL1 | 0.0051210 | 1.49 | 🡹 |
| MICAL3 | 0.0095273 | 1.44 | 🡹 |
| MIOS | 0.0057574 | 1.44 | 🡹 |
| MLLT1 | 0.0010457 | 1.6 | 🡹 |
| MOCS2 | 0.0092615 | 1.36 | 🡹 |
| MRPS22 | 0.0003296 | 1.77 | 🡹 |
| MSH2 | 0.0081745 | 1.28 | 🡹 |
| MSN | 0.0034199 | 1.23 | 🡹 |
| MTCH2 | 0.0055722 | 0.74 | 🡻 |
| MXRA5 | 0.0056097 | 1.37 | 🡹 |
| MYH11 | 0.0003429 | 0.38 | 🡻 |
| MYL6 | 0.0050916 | 1.39 | 🡹 |
| MYO1C | 0.0097531 | 1.32 | 🡹 |
| MYO9B | 0.0015380 | 1.32 | 🡹 |
| NAGK | 0.0001654 | 1.63 | 🡹 |
| NAGLU | 0.0000650 | 1.92 | 🡹 |
| NARF | 0.0010666 | 1.57 | 🡹 |
| NARFL | 0.0043550 | 1.53 | 🡹 |
| NBEAL2 | 0.0035099 | 0.62 | 🡻 |
| NBPF15 | 0.0000027 | 1.82 | 🡹 |
| NBPF15 | 0.0017526 | 1.52 | 🡹 |
| NBPF15 | 0.0018413 | 1.48 | 🡹 |
| NBR1 | 0.0002533 | 1.49 | 🡹 |
| NCK2 | 0.0061640 | 0.45 | 🡻 |
| NCL | 0.0002191 | 0.38 | 🡻 |
| NCL | 0.0021037 | 1.27 | 🡹 |
| NCOA6 | 0.0093784 | 1.31 | 🡹 |
| NDST2 | 0.0069178 | 1.29 | 🡹 |
| NDUFA13 | 0.0008382 | 1.54 | 🡹 |
| NDUFS5 | 0.0015175 | 0.47 | 🡻 |
| NECAP2 | 0.0020689 | 1.36 | 🡹 |
| NELF | 0.0004338 | 1.78 | 🡹 |
| NELF | 0.0028837 | 1.29 | 🡹 |
| NEMF | 0.0067110 | 1.42 | 🡹 |
| NEUROG3 | 0.0011134 | 1.51 | 🡹 |
| NFKB2 | 0.0005887 | 2.25 | 🡹 |
| NFKBID | 0.0004690 | 1.58 | 🡹 |
| NFKBIZ | 0.0015572 | 1.81 | 🡹 |
| NLE1 | 0.0098213 | 1.63 | 🡹 |
| NME2 | 0.0000222 | 1.97 | 🡹 |
| NME2 | 0.0015363 | 1.43 | 🡹 |
| NMT1 | 0.0027944 | 1.42 | 🡹 |
| NOLC1 | 0.0085501 | 1.32 | 🡹 |
| NPEPL1 | 0.0052201 | 1.41 | 🡹 |
| NPIPL3 | 0.0016052 | 1.29 | 🡹 |
| NPLOC4 | 0.0004634 | 1.46 | 🡹 |
| NPM1 | 0.0003781 | 1.44 | 🡹 |
| NPM3 | 0.0048974 | 1.35 | 🡹 |
| NR1H2 | 0.0005348 | 1.46 | 🡹 |
| NR1H2 | 0.0018510 | 1.34 | 🡹 |
| NRBP2 | 0.0086307 | 1.46 | 🡹 |
| NSMCE1 | 0.0073957 | 1.59 | 🡹 |
| NSUN2 | 0.0024077 | 1.61 | 🡹 |
| NT5C | 0.0009126 | 1.5 | 🡹 |
| NUMA1 | 0.0080627 | 1.59 | 🡹 |
| NUP153 | 0.0041007 | 1.52 | 🡹 |
| NUP160 | 0.0007570 | 1.48 | 🡹 |
| NUP160 | 0.0020272 | 1.42 | 🡹 |
| OAS3 | 0.0079915 | 1.44 | 🡹 |
| OGFR | 0.0012485 | 1.41 | 🡹 |
| OTUD1 | 0.0072750 | 1.39 | 🡹 |
| P2RY11 | 0.0016615 | 1.28 | 🡹 |
| PABPC1 | 0.0024337 | 1.39 | 🡹 |
| PCF11 | 0.0000339 | 1.85 | 🡹 |
| PDCD6IP | 0.0000466 | 1.86 | 🡹 |
| PDIA3 | 0.0047856 | 1.5 | 🡹 |
| PDIA3 | 0.0062243 | 1.33 | 🡹 |
| PDLIM2 | 0.0044347 | 1.5 | 🡹 |
| PDPK1 | 0.0001463 | 1.73 | 🡹 |
| PDRG1 | 0.0029424 | 1.41 | 🡹 |
| PDZD4 | 0.0011296 | 1.72 | 🡹 |
| PECAM1 | 0.0095775 | 0.61 | 🡻 |
| PEX7 | 0.0091477 | 1.52 | 🡹 |
| PFKL | 0.0000294 | 1.93 | 🡹 |
| PHAX | 0.0002372 | 1.93 | 🡹 |
| PHC1 | 0.0027223 | 1.71 | 🡹 |
| PHF1 | 0.0025084 | 1.51 | 🡹 |
| PHF3 | 0.0059523 | 1.35 | 🡹 |
| PIDD | 0.0022114 | 1.32 | 🡹 |
| PIK3C2B | 0.0028430 | 1.42 | 🡹 |
| PIK3CD | 0.0006613 | 1.49 | 🡹 |
| PIK3CD | 0.0053767 | 1.36 | 🡹 |
| PIK3CD | 0.0090797 | 1.42 | 🡹 |
| PIK3R2 | 0.0095729 | 1.4 | 🡹 |
| PIM3 | 0.0067098 | 1.39 | 🡹 |
| PIN1 | 0.0022695 | 1.56 | 🡹 |
| PIPSL | 0.0095766 | 1.56 | 🡹 |
| PKD1 | 0.0049951 | 1.58 | 🡹 |
| PKD1L1 | 0.0071400 | 1.42 | 🡹 |
| PKM | 0.0007227 | 1.68 | 🡹 |
| PLCG1 | < 1e-07 | 1.55 | 🡹 |
| PLCG1 | 0.0056365 | 1.28 | 🡹 |
| PLEKHB2 | 0.0051359 | 1.39 | 🡹 |
| PLEKHG2 | 0.0001459 | 1.47 | 🡹 |
| PLIN3 | 0.0015936 | 1.34 | 🡹 |
| PLVAP | 0.0079465 | 1.55 | 🡹 |
| PLXNA1 | 0.0000039 | 1.84 | 🡹 |
| PML | 0.0000981 | 1.34 | 🡹 |
| PMS2 | 0.0009137 | 1.48 | 🡹 |
| PNMA1 | 0.0022037 | 1.36 | 🡹 |
| PNPO | 0.0087477 | 1.45 | 🡹 |
| POLE4 | 0.0082680 | 1.45 | 🡹 |
| POLR1D | 0.0022917 | 1.27 | 🡹 |
| POLR2J | 0.0073627 | 1.38 | 🡹 |
| POLR2L | 0.0027056 | 1.25 | 🡹 |
| POMP | 0.0088723 | 1.2 | 🡹 |
| PPA1 | 0.0004981 | 1.45 | 🡹 |
| PPFIA1 | 0.0000503 | 1.63 | 🡹 |
| PPIF | 0.0034081 | 1.39 | 🡹 |
| PPP1R13L | 0.0015624 | 1.47 | 🡹 |
| PPP1R15A | 0.0032598 | 1.54 | 🡹 |
| PPP1R15A | 0.0070557 | 1.74 | 🡹 |
| PPP1R15B | 0.0069903 | 1.37 | 🡹 |
| PPP1R18 | 0.0067289 | 1.38 | 🡹 |
| PPP1R7 | 0.0052825 | 1.35 | 🡹 |
| PPP1R9B | 0.0000603 | 1.88 | 🡹 |
| PPP5C | 0.0016048 | 1.53 | 🡹 |
| PRDM1 | 0.0000783 | 1.81 | 🡹 |
| PRDX3 | 0.0016025 | 1.42 | 🡹 |
| PRKACA | 0.0000672 | 1.77 | 🡹 |
| PRKAR1A | 0.0024924 | 1.76 | 🡹 |
| PRKCD | 0.0068938 | 1.41 | 🡹 |
| PRKCH | 0.0018063 | 1.36 | 🡹 |
| PRKCZ | 0.0031518 | 1.59 | 🡹 |
| PRMT1 | 0.0032977 | 1.33 | 🡹 |
| PRPF3 | 0.0047775 | 1.65 | 🡹 |
| PRPF4B | 0.0009105 | 1.61 | 🡹 |
| PRPF8 | 0.0015692 | 1.4 | 🡹 |
| PRPF8 | 0.0099337 | 1.27 | 🡹 |
| PRRC1 | 0.0000164 | 1.4 | 🡹 |
| PRRT1 | 0.0002525 | 1.62 | 🡹 |
| PSMB1 | 0.0012705 | 1.69 | 🡹 |
| PSMC4 | 0.0021689 | 1.78 | 🡹 |
| PSMD8 | 0.0047051 | 1.42 | 🡹 |
| PTBP1 | 0.0000167 | 1.57 | 🡹 |
| PTBP1 | 0.0005950 | 1.37 | 🡹 |
| PTBP1 | 0.0031543 | 1.41 | 🡹 |
| PTCD3 | 0.0025711 | 1.27 | 🡹 |
| PTPN1 | 0.0037976 | 1.3 | 🡹 |
| PTPRF | 0.0016523 | 1.55 | 🡹 |
| PTPRK | 0.0000072 | 2.2 | 🡹 |
| RAB14 | 0.0043618 | 1.39 | 🡹 |
| RAB22A | 0.0082527 | 1.41 | 🡹 |
| RAB43 | 0.0049919 | 1.3 | 🡹 |
| RABGGTB | 0.0049840 | 1.38 | 🡹 |
| RAI1 | 0.0027475 | 1.3 | 🡹 |
| RANBP2 | 0.0046561 | 1.26 | 🡹 |
| RAP2C | 0.0093048 | 1.34 | 🡹 |
| RAPGEF1 | 0.0055760 | 1.44 | 🡹 |
| RASA4 | 0.0082384 | 1.41 | 🡹 |
| RASGRP2 | 0.0000060 | 1.79 | 🡹 |
| RASSF1 | 0.0068782 | 1.37 | 🡹 |
| RASSF5 | 0.0075184 | 1.35 | 🡹 |
| RBBP6 | 0.0070404 | 1.33 | 🡹 |
| RBM39 | 0.0073227 | 1.22 | 🡹 |
| RBM5 | 0.0081096 | 1.42 | 🡹 |
| RDBP | 0.0031470 | 1.33 | 🡹 |
| REV3L | 0.0000584 | 1.59 | 🡹 |
| RIOK3 | 0.0069471 | 1.21 | 🡹 |
| RMND1 | 0.0067507 | 1.45 | 🡹 |
| RNF40 | 0.0039213 | 1.42 | 🡹 |
| RPA1 | 0.0038978 | 0.59 | 🡻 |
| RPAP2 | 0.0046579 | 1.33 | 🡹 |
| RPIA | 0.0050211 | 1.49 | 🡹 |
| RPL18 | 0.0086101 | 1.31 | 🡹 |
| RPL19 | 0.0086115 | 1.46 | 🡹 |
| RPL22 | 0.0021997 | 1.4 | 🡹 |
| RPL27A | 0.0001989 | 1.69 | 🡹 |
| RPL28 | 0.0000686 | 1.78 | 🡹 |
| RPL36AL | 0.0087681 | 1.45 | 🡹 |
| RPL37A | 0.0005863 | 1.4 | 🡹 |
| RPL37A | 0.0014723 | 1.57 | 🡹 |
| RPL7 | 0.0000700 | 2.16 | 🡹 |
| RPL7 | 0.0084610 | 1.48 | 🡹 |
| RPS10 | 0.0044003 | 1.34 | 🡹 |
| RPS17 | 0.0036031 | 1.42 | 🡹 |
| RPS17 | 0.0058263 | 1.4 | 🡹 |
| RPS19 | 0.0065846 | 1.31 | 🡹 |
| RPS25 | 0.0056958 | 0.54 | 🡻 |
| RPS27 | 0.0003373 | 1.61 | 🡹 |
| RPS6KB2 | 0.0036961 | 1.44 | 🡹 |
| RPS7 | 0.0005771 | 1.74 | 🡹 |
| RPTOR | 0.0020426 | 1.56 | 🡹 |
| RRS1 | 0.0069495 | 1.38 | 🡹 |
| RSL1D1 | 0.0032687 | 0.62 | 🡻 |
| RSL24D1 | 0.0010766 | 1.43 | 🡹 |
| RSL24D1 | 0.0090462 | 1.35 | 🡹 |
| RUVBL2 | 0.0000021 | 2.03 | 🡹 |
| S1PR4 | 0.0019338 | 1.29 | 🡹 |
| SAMSN1 | 0.0022064 | 1.82 | 🡹 |
| SAP130 | 0.0039745 | 1.52 | 🡹 |
| SBF2 | 0.0005699 | 1.51 | 🡹 |
| SCARF2 | 0.0059093 | 1.65 | 🡹 |
| SEC16A | 0.0050304 | 0.54 | 🡻 |
| SEC62 | 0.0002693 | 1.67 | 🡹 |
| SEPP1 | 0.0078555 | 1.3 | 🡹 |
| SEPT9 | 0.0011764 | 1.68 | 🡹 |
| SERBP1 | 0.0009663 | 1.56 | 🡹 |
| SERBP1 | 0.0026921 | 1.25 | 🡹 |
| SERBP1 | 0.0027297 | 1.4 | 🡹 |
| SERBP1 | 0.0045911 | 1.44 | 🡹 |
| SETD5 | 0.0032644 | 1.53 | 🡹 |
| SFTPA1 | 0.0029737 | 1.62 | 🡹 |
| SFTPA1 | 0.0091904 | 1.39 | 🡹 |
| SFTPB | 0.0035788 | 1.69 | 🡹 |
| SGK2 | 0.0078305 | 1.31 | 🡹 |
| SGSH | 0.0023698 | 1.65 | 🡹 |
| SH2D2A | 0.0001063 | 1.53 | 🡹 |
| SIN3A | 0.0032402 | 1.39 | 🡹 |
| SIPA1L3 | 0.0057248 | 1.42 | 🡹 |
| SKIV2L2 | 0.0006347 | 1.46 | 🡹 |
| SKIV2L2 | 0.0091528 | 0.79 | 🡻 |
| SLC25A29 | 0.0010764 | 1.69 | 🡹 |
| SMC1A | 0.0017383 | 1.49 | 🡹 |
| SMC4 | 0.0008573 | 1.59 | 🡹 |
| SMCHD1 | 0.0031461 | 1.51 | 🡹 |
| SMPD4 | 0.0028919 | 1.64 | 🡹 |
| SNF8 | 0.0001256 | 1.59 | 🡹 |
| SNX17 | 0.0061284 | 1.43 | 🡹 |
| SNX6 | 0.0031276 | 1.3 | 🡹 |
| SORD | 0.0050529 | 1.25 | 🡹 |
| SORD | 0.0059716 | 1.74 | 🡹 |
| SP2 | 0.0002924 | 1.72 | 🡹 |
| SPI1 | 0.0000097 | 1.83 | 🡹 |
| SPNS2 | 0.0068628 | 1.42 | 🡹 |
| SPRY1 | 0.0001899 | 1.76 | 🡹 |
| SPSB3 | 0.0008075 | 1.45 | 🡹 |
| SQRDL | 0.0000725 | 1.49 | 🡹 |
| SRA1 | 0.0025700 | 1.64 | 🡹 |
| SRI | 0.0000554 | 1.67 | 🡹 |
| SRPR | 0.0001654 | 1.35 | 🡹 |
| SRRM2 | 0.0026154 | 1.41 | 🡹 |
| SRRM2 | 0.0064665 | 1.28 | 🡹 |
| SRSF3 | 0.0024119 | 0.54 | 🡻 |
| SSH3 | 0.0022999 | 1.4 | 🡹 |
| SSR2 | 0.0030130 | 1.46 | 🡹 |
| SSRP1 | 0.0031840 | 1.5 | 🡹 |
| ST3GAL3 | 0.0046731 | 0.36 | 🡻 |
| STAT1 | 0.0037186 | 1.32 | 🡹 |
| STAT3 | 0.0077622 | 1.37 | 🡹 |
| STIM2 | 0.0051122 | 1.35 | 🡹 |
| STIP1 | 0.0037905 | 1.47 | 🡹 |
| STMN1 | 0.0004167 | 0.41 | 🡻 |
| STUB1 | 0.0005099 | 0.47 | 🡻 |
| SUOX | 0.0034802 | 1.55 | 🡹 |
| SURF6 | 0.0022498 | 1.44 | 🡹 |
| SYNRG | 0.0007450 | 1.38 | 🡹 |
| SYTL1 | 0.0020760 | 1.77 | 🡹 |
| TACC2 | 0.0001634 | 0.45 | 🡻 |
| TAF2 | 0.0032924 | 1.46 | 🡹 |
| TAF4B | 0.0035117 | 1.51 | 🡹 |
| TAGLN3 | 0.0004579 | 2.11 | 🡹 |
| TAOK2 | 0.0091711 | 1.42 | 🡹 |
| TARS | 0.0037477 | 1.48 | 🡹 |
| TBCC | 0.0012148 | 1.38 | 🡹 |
| TBCC | 0.0048387 | 0.53 | 🡻 |
| TBCD | 0.0042073 | 1.36 | 🡹 |
| TBX21 | 0.0040420 | 1.41 | 🡹 |
| TCEA1 | 0.0023872 | 1.51 | 🡹 |
| TCF3 | 0.0024388 | 1.41 | 🡹 |
| TCF3 | 0.0090074 | 1.61 | 🡹 |
| TGOLN2 | 0.0080647 | 1.3 | 🡹 |
| THAP4 | 0.0016495 | 1.5 | 🡹 |
| THBD | 0.0001397 | 1.65 | 🡹 |
| THBS1 | < 1e-07 | 3 | 🡹 |
| THBS3 | 0.0054315 | 1.36 | 🡹 |
| THOC2 | 0.0004409 | 1.67 | 🡹 |
| TLE1 | 0.0000009 | 2.05 | 🡹 |
| TLK2 | 0.0014608 | 1.32 | 🡹 |
| TLN1 | 0.0000639 | 1.89 | 🡹 |
| TMC8 | 0.0000149 | 2.27 | 🡹 |
| TMEM2 | 0.0001079 | 1.54 | 🡹 |
| TNFAIP2 | 0.0047987 | 1.61 | 🡹 |
| TNFAIP8 | 0.0009237 | 1.41 | 🡹 |
| TNFAIP8 | 0.0039832 | 1.61 | 🡹 |
| TNFAIP8 | 0.0079094 | 1.44 | 🡹 |
| TNFRSF4 | 0.0054238 | 1.43 | 🡹 |
| TNRC18 | 0.0030357 | 1.56 | 🡹 |
| TNRC6B | 0.0093849 | 1.27 | 🡹 |
| TNS3 | 0.0000293 | 2.04 | 🡹 |
| TONSL | 0.0099974 | 1.3 | 🡹 |
| TP53 | 0.0001362 | 1.75 | 🡹 |
| TPI1 | 0.0073693 | 1.33 | 🡹 |
| TPX2 | 0.0000020 | 2.95 | 🡹 |
| TRAF1 | 0.0014886 | 1.48 | 🡹 |
| TREX1 | 0.0095576 | 1.38 | 🡹 |
| TRIM22 | 0.0057319 | 1.34 | 🡹 |
| TRIM28 | 0.0060872 | 1.61 | 🡹 |
| TRIM44 | 0.0013228 | 1.67 | 🡹 |
| TRIM78P | 0.0007562 | 1.79 | 🡹 |
| TRIOBP | 0.0024528 | 1.52 | 🡹 |
| TSC2 | 0.0023300 | 1.42 | 🡹 |
| TSR1 | 0.0073309 | 1.32 | 🡹 |
| TSTA3 | 0.0090939 | 0.77 | 🡻 |
| TTLL1 | 0.0013927 | 0.46 | 🡻 |
| TUBB3 | 0.0006225 | 1.49 | 🡹 |
| TUBGCP2 | 0.0022412 | 1.57 | 🡹 |
| TXNDC5 | 0.0023195 | 1.37 | 🡹 |
| UBA1 | 0.0020506 | 1.45 | 🡹 |
| UBB | 0.0081364 | 1.47 | 🡹 |
| UBE2A | 0.0039039 | 1.56 | 🡹 |
| UBE2N | 0.0025555 | 1.58 | 🡹 |
| UBR4 | 0.0044353 | 1.51 | 🡹 |
| UBXN1 | 0.0069179 | 1.42 | 🡹 |
| UBXN4 | 0.0021558 | 1.31 | 🡹 |
| UBXN6 | 0.0011168 | 1.66 | 🡹 |
| UIMC1 | 0.0053052 | 1.28 | 🡹 |
| ULK1 | 0.0079923 | 1.49 | 🡹 |
| UMPS | 0.0000024 | 2.05 | 🡹 |
| UNC13D | 0.0001264 | 1.72 | 🡹 |
| UROD | 0.0039035 | 0.7 | 🡻 |
| USP36 | 0.0046025 | 1.37 | 🡹 |
| USP7 | 0.0066276 | 1.42 | 🡹 |
| UTP14A | 0.0027308 | 1.52 | 🡹 |
| VAT1 | 0.0000725 | 0.34 | 🡻 |
| VAT1 | 0.0052251 | 1.38 | 🡹 |
| VAV1 | 0.0049817 | 1.46 | 🡹 |
| VCAN | 0.0003054 | 1.68 | 🡹 |
| VCPIP1 | 0.0058068 | 1.5 | 🡹 |
| VIM | 0.0047357 | 1.48 | 🡹 |
| VIMP | 0.0091528 | 1.39 | 🡹 |
| WASH1 | 0.0013424 | 0.47 | 🡻 |
| WASH1 | 0.0074159 | 1.39 | 🡹 |
| WASL | 0.0034431 | 1.49 | 🡹 |
| WDR13 | 0.0095082 | 1.37 | 🡹 |
| WDR73 | 0.0008074 | 1.51 | 🡹 |
| WDR74 | 0.0018082 | 1.3 | 🡹 |
| WDR74 | 0.0057339 | 1.29 | 🡹 |
| WNK2 | 0.0072607 | 1.34 | 🡹 |
| XPNPEP3 | 0.0018222 | 1.35 | 🡹 |
| YEATS2 | 0.0008726 | 1.49 | 🡹 |
| YTHDC1 | 0.0068757 | 1.28 | 🡹 |
| YWHAE | 0.0049741 | 1.55 | 🡹 |
| YWHAZ | 0.0002277 | 1.6 | 🡹 |
| ZBP1 | 0.0015778 | 1.63 | 🡹 |
| ZC3H13 | 0.0059352 | 1.23 | 🡹 |
| ZC3H3 | 0.0082995 | 1.3 | 🡹 |
| ZCCHC3 | 0.0001965 | 1.6 | 🡹 |
| ZFP36L1 | 0.0045734 | 1.58 | 🡹 |
| ZGPAT | 0.0063147 | 1.37 | 🡹 |
| ZMYM2 | 0.0063778 | 1.71 | 🡹 |
| ZNF260 | 0.0065529 | 1.36 | 🡹 |
| ZNF335 | 0.0019070 | 1.46 | 🡹 |
| ZNF335 | 0.0093957 | 1.26 | 🡹 |
| ZNF341 | 0.0081258 | 1.36 | 🡹 |
| ZNF358 | 0.0079232 | 0.57 | 🡻 |
| ZNF384 | 0.0094322 | 1.36 | 🡹 |
| ZNF428 | 0.0000065 | 1.9 | 🡹 |
| ZNF440 | 0.0043430 | 1.37 | 🡹 |
| ZNF652 | 0.0033274 | 1.29 | 🡹 |
| ZNF668 | 0.0050944 | 1.49 | 🡹 |
| ZNF672 | 0.0000753 | 1.45 | 🡹 |
| ZNF7 | 0.0000254 | 2.06 | 🡹 |
| ZNF76 | 0.0000589 | 1.59 | 🡹 |
| ZNF761 | 0.0005440 | 1.65 | 🡹 |
| ZNF777 | 0.0005106 | 1.53 | 🡹 |
| ZNF837 | 0.0089269 | 1.28 | 🡹 |
| ZNFX1 | 0.0011730 | 1.54 | 🡹 |
