# Supplementary material for: The Immunome of Colon Cancer: Functional In Silico Analysis of Antigenic Proteins Deduced from IgG Microarray Profiling
Source: Genomics Proteomics Bioinformatics. 2018 Mar 2;16(1):73–84. doi: 10.1016/j.gpb.2017.10.002 (PMC6000238; doi:10.1016/j.gpb.2017.10.002)
Supplement: Supplementary Table S2 — Complete list of 50 pathways enriched with DIRAGs analyzed using IPA (P < 0.01) [file mmc4.docx]

**Table S2 Complete list of 50 pathways enriched with DIRAGs analyzed using IPA (*P* < 0.01)**

| **Pathway** | **-Log (*P* value)** | **Ratio** | **Proteins** |
| --- | --- | --- | --- |
| EIF2 signaling | 5.39 | 0.16 | PABPC1, PIK3C2B, RPL22, RPL27A, RPL37A, RPS19, PDPK1, PPP1R15A, RPS17/RPS17L, EIF4G1, RPL7, RPS7, EIF3G, EIF3F, RPS27, EIF4G2, RPL28, RPL36AL, RPL19, RPS25, PIK3CD, PIK3R2, RPS10, RPL18 |
| mTOR signaling | 4.63 | 0.15 | PIK3C2B, ULK1, DDIT4, RPS19, PDPK1, RPS17/RPS17L, EIF4G1, PRKCZ, EIF3G, RPS7, DGKZ, EIF3F, RPS27, EIF4G2, PRKCD, TSC2, RPS6KB2, RPTOR, RPS25, PRKCH, PIK3CD, PIK3R2, RPS10 |
| Growth hormone signaling | 4.41 | 0.23 | PIK3C2B, PRKCD, RPS6KB2, PLCG1, PDPK1, PRKCH, PIK3CD, STAT3, PIK3R2, STAT1, ELK1, PRKCZ |
| Virus entry via endocytic pathways | 3.71 | 0.18 | PIK3C2B, FLNB, AP1G2, HLA-C, HLA-A, PRKCD, CLTA, HLA-B, PLCG1, PIK3CD, PRKCH, PIK3R2, PRKCZ |
| 14-3-3-mediated signaling | 3.45 | 0.16 | PIK3C2B, TUBB3, WHAE, PDIA3, YWHAZ, PLCG1, VIM, PRKCZ, RKCD, TSC2, PIK3CD, PRKCH, PIK3R2, ELK1, PDCD6IP |
| Signaling by Rho family GTPases | 3.29 | 0.13 | PIK3C2B, MYL6, SEPT9, VIM, ARHGEF1, NFKB2, IQGAP1, CLIP1, PRKCZ, CDH2, STMN1, ARFIP2, WASL, ARHGEF16, GNAO1, GNB2, PIK3CD, PIK3R2, ARHGEF3, ELK1, MSN |
| Calcium-induced T lymphocyte apoptosis | 3.14 | 0.23 | LCK, PRKCD, HDAC1, PLCG1, CAPN2, PRKCH, PRKCZ, CABIN1 |
| HGF signaling | 3.14 | 0.16 | ETS1, PIK3C2B, RAPGEF1, PRKCD, PLCG1, PIK3CD, PRKCH, PIK3R2, STAT3, ELK1, CCND1, PRKCZ, ELF1 |
| Thrombopoietin signaling | 3.09 | 0.21 | PIK3C2B, PRKCD, PLCG1, PRKCH, PIK3CD, STAT3, PIK3R2, STAT1, PRKCZ |
| HER-2 signaling in breast cancer | 3.05 | 0.18 | TP53, PIK3C2B, PRKCD, TSC2, PLCG1, ERBB3, PRKCH, PIK3CD, PIK3R2, CCND1, PRKCZ |
| Erythropoietin signaling | 2.89 | 0.18 | PIK3C2B, PRKCD, PLCG1, PDPK1, PRKCH, PIK3CD, PIK3R2, NFKB2, ELK1, PRKCZ |
| Endometrial cancer signaling | 2.88 | 0.19 | TP53, PIK3C2B, AXIN1, CTNNA1, PDPK1, PIK3CD, PIK3R2, ELK1, CCND1 |
| Glycolysis I | 2.78 | 0.31 | GPI, TPI1, PKM, PFKL, ALDOC |
| IL-3 signaling | 2.77 | 0.17 | RAPGEF1, PIK3C2B, PRKCD, PRKCH, PIK3CD, STAT3, PIK3R2, STAT1, ELK1, PRKCZ |
| Non-small cell lung cancer signaling | 2.71 | 0.17 | TP53, PIK3C2B, PLCG1, PDPK1, PIK3CD, RASSF5, PIK3R2, CCND1, SIN3A, RASSF1 |
| Regulation of eIF4 and p70S6K signaling | 2.67 | 0.13 | PABPC1, PIK3C2B, RPS19, PDPK1, RPS17/RPS17L, EIF4G1, PRKCZ, EIF3G, RPS7, EIF3F, RPS27, EIF4G2, RPS25, PIK3CD, PIK3R2, RPS10 |
| Huntington's disease signaling | 2.67 | 0.12 | TP53, PIK3C2B, UBB, HSPA1A/HSPA1B, HDAC1, POLR2J, PDPK1, SIN3A, PRKCZ, POLR2L, ARFIP2, PRKCD, GNB2, CASP1, CAPN2, PRKCH, PIK3CD, PIK3R2, GOSR1, CASP8 |
| Insulin receptor signaling | 2.65 | 0.14 | PIK3C2B, RAPGEF1, GYS1, PPP1R7, PTPN1, TSC2, RPTOR, RPS6KB2, PDPK1, PIK3CD, PIK3R2, PRKCZ, PTPRF, PRKAR1A |
| Thrombin signaling | 2.65 | 0.13 | PIK3C2B, MYL6, PDIA3, PLCG1, PDPK1, ARHGEF1, NFKB2, PRKCZ, ARHGEF16, PRKCD, GNAO1, GNB2, PRKCH, PIK3CD, PIK3R2, ARHGEF3, ELK1 |
| Neuropathic pain signaling in dorsal horn neurons | 2.6 | 0.16 | PIK3C2B, PDIA3, PRKCD, PLCG1, PRKCH, PIK3CD, PIK3R2, ELK1, PRKCZ, PRKAR1A |
| Prolactin signaling | 2.6 | 0.16 | PIK3C2B, PRKCD, PLCG1, PDPK1, PRKCH, PIK3CD, STAT3, PIK3R2, STAT1, PRKCZ |
| Renin-angiotensin signaling | 2.58 | 0.15 | PIK3C2B, PRKCD, PLCG1, PRKCH, PIK3CD, STAT3, PIK3R2, NFKB2, STAT1, ELK1, PRKCZ, PRKAR1A |
| Systemic lupus erythematosus signaling | 2.54 | 0.13 | LSM14A, PIK3C2B, LSM14B, LCK, HLA-C, PRPF3, HLA-A, PRPF4B, PRPF8, HLA-B, HNRNPA2B1, PLCG1, PIK3CD, PIK3R2, HNRNPC |
| Androgen signaling | 2.54 | 0.15 | POLR2L, PRKCD, ERCC3, GNAO1, POLR2J, GNB2, PRKCH, NFKB2, CCND1, TAF2, PRKCZ, PRKAR1A |
| ERK/MAPK signaling | 2.52 | 0.12 | ETS1, PIK3C2B, RAPGEF1, YWHAZ, PLCG1, TLN1, STAT3, ELF1, KSR1, PPP1R7, PRKCD, PIK3CD, PIK3R2, STAT1, ELK1, JMJD7-PLA2G4B, PRKAR1A |
| Tec kinase signaling | 2.46 | 0.13 | PIK3C2B, PLCG1, STAT3, NFKB2, PRKCZ, LCK, PRKCD, GNAO1, GNB2, PIK3CD, VAV1, PRKCH, PIK3R2, STAT1 |
| Ovarian cancer signaling | 2.41 | 0.13 | TP53, PIK3C2B, PMS2, MSH2, AXIN1, RPS6KB2, PIK3CD, PIK3R2, TCF3, CCND1, SIN3A, APC, PRKAR1A |
| Role of p14/p19ARF in tumor suppression | 2.41 | 0.22 | TP53, NPM1, PIK3C2B, NPM3, PIK3CD, PIK3R2 |
| Glioma signaling | 2.37 | 0.15 | TP53, PIK3C2B, E2F4, PRKCD, PLCG1, PRKCH, PIK3CD, PIK3R2, CCND1, PRKCZ, SIN3A |
| IL-9 signaling | 2.33 | 0.21 | PIK3C2B, PIK3CD, STAT3, PIK3R2, NFKB2, STAT1 |
| ErbB signaling | 2.25 | 0.15 | PIK3C2B, PRKCD, PLCG1, PDPK1, ERBB3, PRKCH, PIK3CD, PIK3R2, ELK1, PRKCZ |
| ErbB4 signaling | 2.23 | 0.17 | PIK3C2B, PRKCD, PLCG1, PDPK1, PRKCH, PIK3CD, PIK3R2, PRKCZ |
| Hereditary breast cancer signaling | 2.22 | 0.13 | POLR2L, TP53, PIK3C2B, NPM1, PMS2, UBB, MSH2, HDAC1, POLR2J, PIK3CD, RPA1, PIK3R2, CCND1 |
| Breast cancer regulation by stathmin1 | 2.17 | 0.12 | TP53, PIK3C2B, TUBB3, E2F4, ARHGEF1, PRKCZ, STMN1, PPP1R7, PRKCD, ARHGEF16, GNB2, PRKCH, PIK3CD, PIK3R2, ARHGEF3, PRKAR1A |
| ErbB2-ErbB3 signaling | 2.17 | 0.16 | PIK3C2B, PDPK1, ERBB3, PIK3CD, STAT3, PIK3R2, ELK1, CCND1 |
| p70S6K signaling | 2.17 | 0.13 | PIK3C2B, IL4R, YWHAE, PDIA3, PRKCD, YWHAZ, PLCG1, PDPK1, PRKCH, PIK3CD, PIK3R2, PRKCZ |
| CREB signaling in neurons | 2.14 | 0.12 | POLR2L, PIK3C2B, PDIA3, PRKCD, GNAO1, GNB2, POLR2J, PLCG1, PRKCH, PIK3CD, PIK3R2, ELK1, PRKCZ, PRKAR1A |
| Aldosterone signaling in epithelial cells | 2.12 | 0.12 | PIK3C2B, HSP90AB1, PDIA3, HSPA1A/HSPA1B, PRKCD, PDPK1, PLCG1, PIK3CD, PRKCH, DNAJC10, PIK3R2, DNAJA1, PRKCZ |
| GM-CSF signaling | 2.12 | 0.16 | ETS1, PIK3C2B, PIK3CD, STAT3, PIK3R2, STAT1, ELK1, CCND1 |
| PI3K/AKT signaling | 2.08 | 0.12 | TP53, YWHAE, YWHAZ, PDPK1, NFKB2, CCND1, PRKCZ, GYS1, HSP90AB1, TSC2, RPS6KB2, PIK3CD, PIK3R2 |
| Neuregulin signaling | 2.08 | 0.14 | HSP90AB1, PRKCD, RPS6KB2, PLCG1, PDPK1, ERBB3, PRKCH, PIK3R2, ELK1, PRKCZ |
| Estrogen-dependent breast cancer signaling | 2.06 | 0.16 | PIK3C2B, PIK3CD, PIK3R2, NFKB2, AKR1C4, ELK1, CCND1, HSD17B4 |
| P2Y purigenic receptor signaling pathway | 2.04 | 0.13 | PIK3C2B, PDIA3, PRKCD, GNB2, PLCG1, PRKCH, PIK3CD, PIK3R2, NFKB2, PRKCZ, PRKAR1A |
| Role of pattern recognition receptors in recognition of bacteria and viruses | 2.04 | 0.15 | PIK3C2B, PRKCD, CASP1, PRKCH, PIK3CD, PIK3R2, NFKB2, OAS3, PRKCZ |
| Acute myeloid leukemia signaling | 2.04 | 0.15 | PIK3C2B, RPS6KB2, PIK3CD, STAT3, PIK3R2, NFKB2, PML, TCF3, CCND1 |
| Prostate cancer signaling | 2.03 | 0.14 | TP53, PIK3C2B, HSP90AB1, PDPK1, PIK3CD, PIK3R2, NFKB2, CCND1, GSTP1, SIN3A |
| Myc mediated apoptosis signaling | 2.01 | 0.15 | TP53, PIK3C2B, YWHAE, YWHAZ, PIK3CD, PIK3R2, CASP8, PRKCZ |
| Activation of IRF by cytosolic pattern recognition receptors | 2.01 | 0.17 | ZBP1, IRF9, PIN1, NFKB2, STAT1, ADAR, ISG15 |
| UVB-induced MAPK signaling | 2.01 | 0.17 | TP53, PIK3C2B, PRKCD, PRKCH, PIK3CD, PIK3R2, PRKCZ |
| IGF-1 signaling | 2.01 | 0.13 | PIK3C2B, YWHAE, YWHAZ, RPS6KB2, PDPK1, PIK3CD, STAT3, PIK3R2, ELK1, PRKCZ, PRKAR1A |

*Note*: The ratio is the number of proteins in a given pathway that meet the cutoff criteria (*P* < 0.01), divided by the total number of proteins that make up that pathway.
