# Supplementary material for: The Immunome of Colon Cancer: Functional In Silico Analysis of Antigenic Proteins Deduced from IgG Microarray Profiling
Source: Genomics Proteomics Bioinformatics. 2018 Mar 2;16(1):73–84. doi: 10.1016/j.gpb.2017.10.002 (PMC6000238; doi:10.1016/j.gpb.2017.10.002)
Supplement: Supplementary Table S3 — Top 5 network functions and the associated DIRAGs analyzed using IPA [file mmc5.docx]

**Table S3 Top 5 network functions and the associated DIRAGs analyzed using IPA**

| **Associated network functions** | **DIRAGs found in the network** |
| --- | --- |
| Cell death and survival, cell cycle, cellular growth and proliferation | BHLHE40; CCND1; CHD3; CTNND1; DAXX; GNL3; HMGN2; HSPA1A/HSPA1B; ISG15; JUNB; KLF6; KSR1; LGALS3; NCL; NPM1; NUP153; PDCD6IP; PHF1; PML; PPIF; PPP5C; PRKCZ; PRPF8; PTPN1; RAP2C; RBBP6; SEC62; SRSF3; STMN1; TP53; TSC2; UBE2N; UIMC1; VCAN; ZFP36L1 |
| Cellular movement, cellular growth and proliferation, cell cycle | ALDOC; CDH2; CPE; ELF1; ELK1; EPHB3; ERBB3; FLNB; GLUL; LRIG1; PKM; PLCG1; STAT1; STAT3; THBS1; VIM |
| Cell cycle, cellular development, cellular growth and proliferation | CCND1; CCT5; CCT6A; CTNNA1; DNAJA1; DNAJA2; DYNC1H1; E2F4; GDI1; IRF9; KLF6; KRT8; LMNA; NFKB2; NUMA1; PKD1; RASSF1; TRIM28 |
| Cell death and survival, cell cycle, cellular development | ARID1A; BAG1; BAG6; BCLAF1; HDAC1; KLF2; KPNA2; LAMA5; LDB1; NAGK; NCL; PIN1; PP1R15A; PRDM1; SMC4; ST3GAL3; UROD |
| Cell death and survival, cancer, reproductive system disease | ADAM8; AHNAK; APC; BHLHE40; CCND1; DDIT4; EHMT2; IMPDH2; IQGAP1; LARS; LRP1; PPP1R13L; RANBP2; TP53; TRIOBP |
