# Supplementary material for: The Immunome of Colon Cancer: Functional In Silico Analysis of Antigenic Proteins Deduced from IgG Microarray Profiling
Source: Genomics Proteomics Bioinformatics. 2018 Mar 2;16(1):73–84. doi: 10.1016/j.gpb.2017.10.002 (PMC6000238; doi:10.1016/j.gpb.2017.10.002)
Supplement: Supplementary Table S4 — List of antigens overlapping between DIRAGs identified in this study and SEREX antigens [file mmc6.docx]

**Table S4 List of antigens overlapping between DIRAGs identified in this study and SEREX antigens**

| [ACADVL](http://ludwig-sun5.unil.ch/CancerImmunomeDB/ShowGene.php?Gene=ACADVL) | DNAJA1 | MSN | RPS27 |
| --- | --- | --- | --- |
| ACIN1 | DNAJA2 | MYH11 | RSL1D1 |
| ANKRD11 | EEF1D | MYO9B | RUVBL2 |
| [ANKRD12](http://ludwig-sun5.unil.ch/CancerImmunomeDB/ShowGene.php?Gene=ANKRD12) | EIF4G2 | NAGK | SCARF2 |
| AP1G2 | EPHB3 | NARF | SIN3A |
| AP3D1 | ERBB3 | NCOA6 | [SNX6](http://ludwig-sun5.unil.ch/CancerImmunomeDB/ShowGene.php?Gene=SNX6) |
| ARCN1 | [EXOSC8](http://ludwig-sun5.unil.ch/CancerImmunomeDB/ShowGene.php?Gene=EXOSC8) | NDUFA13 | SSRP1 |
| [ARID5A](http://ludwig-sun5.unil.ch/CancerImmunomeDB/ShowGene.php?Gene=ARID5A) | FAM48A | NME2 | STIP1 |
| ARPC1B | FBXO7 | NSUN2 | STUB1 |
| ASPSCR1 | GBP5 | OGFR | SURF6 |
| AURKAIP1 | GCC2 | [PABPC1](http://ludwig-sun5.unil.ch/CancerImmunomeDB/ShowGene.php?Gene=PABPC1) | [TACC2](http://ludwig-sun5.unil.ch/CancerImmunomeDB/ShowGene.php?Gene=TACC2) |
| BAZ1A | GNL3 | PHF3 | TCF3 |
| BCR | GOSR1 | PKD1 | THOC2 |
| BRD3 | [GRASP](http://ludwig-sun5.unil.ch/CancerImmunomeDB/ShowGene.php?Gene=GRASP) | PPP1R15A | TLN1 |
| C9orf86 | HIP1R | PRKAR1A | [TNRC18](http://ludwig-sun5.unil.ch/CancerImmunomeDB/ShowGene.php?Gene=TNRC18) |
| CCNL1 | HK1 | PTBP1 | TNRC6B |
| CCT5 | [HLA-B](http://ludwig-sun5.unil.ch/CancerImmunomeDB/ShowGene.php?Gene=HLA-B) | RAI1 | [TPI1](http://ludwig-sun5.unil.ch/CancerImmunomeDB/ShowGene.php?Gene=TPI1) |
| CCT6A | [HLA-C](http://ludwig-sun5.unil.ch/CancerImmunomeDB/ShowGene.php?Gene=HLA-C) | RANBP2 | TPX2 |
| COL3A1 | HMG20B | [RASSF1](http://ludwig-sun5.unil.ch/CancerImmunomeDB/ShowGene.php?Gene=RASSF1) | TREX1 |
| CPE | HMGN2 | RBBP6 | UBB |
| CPNE1 | IL16 | RBM5 | UTP14A |
| CTNNA1 | [IVNS1ABP](http://ludwig-sun5.unil.ch/CancerImmunomeDB/ShowGene.php?Gene=IVNS1ABP) | RDBP | VIM |
| CYHR1 | KRT8 | RNF40 | WNK2 |
| DBNL | [LMNA](http://ludwig-sun5.unil.ch/CancerImmunomeDB/ShowGene.php?Gene=LMNA) | RPL18 | [YWHAE](http://ludwig-sun5.unil.ch/CancerImmunomeDB/ShowGene.php?Gene=YWHAE) |
| DDX21 | LYAR | [RPL27A](http://ludwig-sun5.unil.ch/CancerImmunomeDB/ShowGene.php?Gene=RPL27A) | YWHAZ |
| DHX9 | MAZ | RPL37A |  |
