# Supplementary material for: The Immunome of Colon Cancer: Functional In Silico Analysis of Antigenic Proteins Deduced from IgG Microarray Profiling
Source: Genomics Proteomics Bioinformatics. 2018 Mar 2;16(1):73–84. doi: 10.1016/j.gpb.2017.10.002 (PMC6000238; doi:10.1016/j.gpb.2017.10.002)
Supplement: Supplementary Table S5 — Compilation of CRC TAAs from literature [file mmc7.docx]

**Table S5 Compilation of CRC TAAs from literature**

| **No.** | **TAA** | **Refs.** | **PMID** | **No.** | **TAA** | **Refs.** | **PMID** | **No.** | **TAA** | **Refs.** | **PMID** |
| --- | --- | --- | --- | --- | --- | --- | --- | --- | --- | --- | --- |
| **1** | A0PJ75 | [31] | 19828471 | **45** | ITGA6 | [32] | 19638618 | **89** | RPS6KA2 | [32] | 19638618 |
| **2** | ACVR2B | [5,32] | 22465712; 19638618 | **46** | KDR | [32] | 19638618 | **90** | SALL2 | [32] | 19638618 |
| **3** | ADSL | [44] | 21137041 | **47** | KIAA1416 | [43,44] | 12124339; 21137041 | **91** | SCP2 | [44] | 21137041 |
| **4** | AGBL5 | [44] | 21137041 | **48** | KIF2C | [44] | 21137041 | **92** | SDCCAG1 | [44] | 21137041 |
| **5** | AKT1 | [32] | 19638618 | **49** | KNSL6 | [43] | 12124339 | **93** | SDCCAG10 | [44] | 21137041 |
| **6** | BAC85857 | [31] | 19828471 | **50** | Koc | [41] | 19140877 | **94** | SDCCAG3 | [44] | 21137041 |
| **7** | BHMT2 | [32] | 19638618 | **51** | LASS5 | [31] | 19828471 | **95** | SDCCAG8 | [44] | 21137041 |
| **8** | BMX | [32] | 19638618 | **52** | LGR6 | [44] | 21137041 | **96** | Seb4D | [43] | 12124339 |
| **9** | C21orf2 | [44] | 21137041 | **53** | LIMS1 | [44] | 21137041 | **97** | SEC16A | [31] | 19828471 |
| **10** | C6orf192 | [44] | 21137041 | **54** | LMNA | [43] | 12124339 | **98** | SNP29 | [31] | 19828471 |
| **11** | C9orf43 | [32] | 19638618 | **55** | LMTK2 | [44] | 21137041 | **99** | SRC | [32] | 19638618 |
| **12** | CADM1 | [31] | 19828471 | **56** | MAGEA3 | [43,44] | 12124339; 21137041 | **100** | SREBF2 | [5] | 22465712 |
| **13** | Cbx5 | [44] | 21137041 | **57** | MAPKAPK3 | [32] | 19638618 | **101** | SSRP1 | [44] | 21137041 |
| **14** | CEA | [42] | 8758246 | **58** | MAPKAPK5 | [32] | 19638618 | **102** | SSX2 | [43] | 12124339 |
| **15** | CLNS1A | [31] | 19828471 | **59** | MBD2 | [43,44] | 12124339; 21137041 | **103** | STARD10 | [44] | 21137041 |
| **16** | c-myc | [41] | 19140877 | **60** | MFAP2 | [32] | 19638618 | **104** | STAU1 | [32] | 19638618 |
| **17** | COASY | [32] | 19638618 | **61** | MKNK1 | [44] | 21137041 | **105** | STK4 | [32] | 19638618 |
| **18** | CSNK1G2 | [32] | 19638618 | **62** | NAP1L1 | [44] | 21137041 | **106** | STOM | [31] | 19828471 |
| **19** | CTAG1A | [44] | 21137041 | **63** | NEK3 | [32] | 19638618 | **107** | STUB1 | [44] | 21137041 |
| **20** | DAPK1 | [32] | 19638618 | **64** | NFYA | [32] | 19638618 | **108** | TAF10 | [44] | 21137041 |
| **21** | DUSP8 | [31] | 19828471 | **65** | NHSL1 | [5] | 22465712 | **109** | TAX1BP1 | [44] | 21137041 |
| **22** | EFNA3 | [32] | 19638618 | **66** | NMDAR | [45] | 19795454 | **110** | TCF3 | [31] | 19828471 |
| **23** | EPRS | [44] | 21137041 | **67** | NOXA1 | [44] | 21137041 | **111** | TDRD6 | [44] | 21137041 |
| **24** | FA59A | [31] | 19828471 | **68** | NR_003287 | [31] | 19828471 | **112** | TP53 | [31,41, 43-45] | 19828471; 19140877; 12124339; 21137041;  19795454 |
| **25** | FAS | [44] | 21137041 | **69** | NUCB1 | [44] | 21137041 | **113** | TPM4 | [32] | 19638618 |
| **26** | FGFR4 | [5,32] | 22465712; 19638618 | **70** | NY-CO-16 | [45] | 19795454 | **114** | TRIM21 | [32] | 19638618 |
| **27** | FLJ10154 | [31] | 19828471 | **71** | NY-CO-41 | [44] | 21137041 | **115** | TRIM28 | [31] | 19828471 |
| **28** | GOLGA4 | [44] | 21137041 | **72** | NY-CO-45 | [43] | 12124339 | **116** | TRIP4 | [43,44] | 12124339; 21137041 |
| **29** | GOLGB1 | [44] | 21137041 | **73** | NY-CO-8 | [45] | 19795454 | **117** | TSHZ1 | [44] | 21137041 |
| **30** | GRK7 | [32] | 19638618 | **74** | NY-ESO-1 | [43] | 12124339 | **118** | TSLC1 | [31] | 19828471 |
| **31** | HDAC1 | [32] | 19638618 | **75** | p62 | [41] | 19140877 | **119** | TSLP | [32] | 19638618 |
| **32** | HDAC5 | [43,45] | 12124339; 19795454 | **76** | PBK | [32] | 19638618 | **120** | TTLL7 | [32] | 19638618 |
| **33** | HIP1R | [43] | 12124339 | **77** | PDE4A | [44] | 21137041 | **121** | UBE3A | [44] | 21137041 |
| **34** | HMGB1 | [31] | 19828471 | **78** | PDGFRB | [32] | 19638618 | **122** | USH1C | [44] | 21137041 |
| **35** | HMGN2 | [44] | 21137041 | **79** | PDXK | [44] | 21137041 | **123** | VGLL4 | [31] | 19828471 |
| **36** | HMMR | [44] | 21137041 | **80** | PFDN5 | [32] | 19638618 | **124** | WBP2 | [32] | 19638618 |
| **37** | HNRDL | [31] | 19828471 | **81** | PIM1 | [5,44] | 22465712; 21137041 | **125** | ZEP1 | [32] | 19828471 |
| **38** | HSPA4 | [44] | 21137041 | **82** | PKN1 | [32] | 19638618 | **126** | Znf292 | [44] | 21137041 |
| **39** | HSPH1 | [44] | 21137041 | **83** | PKN2 | [32] | 19638618 | **127** | ZNF346 | [31] | 19828471 |
| **40** | ICLN | [31] | 19828471 | **84** | PRKCD | [32] | 19638618 | **128** | ZNF638 | [31] | 19828471 |
| **41** | IGLC1 | [32] | 19638618 | **85** | RBMS1 | [44] | 21137041 | **129** | ZNF700 | [31] | 19828471 |
| **42** | Imp1 | [41] | 19140877 | **86** | RBPJ | [32] | 19638618 | **130** | ZNF706 | [32] | 19638618 |
| **43** | IRAK4 | [32] | 19638618 | **87** | RIOK1 | [44] | 21137041 | **131** | ZNF768 | [31] | 19828471 |
| **44** | ITFG3 | [31] | 19828471 | **88** | RPS6KA1 | [32] | 19638618 |  |  |  |  |
