# Supplementary material for: The Immunome of Colon Cancer: Functional In Silico Analysis of Antigenic Proteins Deduced from IgG Microarray Profiling
Source: Genomics Proteomics Bioinformatics. 2018 Mar 2;16(1):73–84. doi: 10.1016/j.gpb.2017.10.002 (PMC6000238; doi:10.1016/j.gpb.2017.10.002)
Supplement: Supplementary Table S6 — List of CRC-associated genes [file mmc8.docx]

**Table S6 List of CRC-associated genes**

| **Gene** | **Refs.** | **PMID** | **Gene** | **Refs.** | **PMID** |
| --- | --- | --- | --- | --- | --- |
| *AKT1* | [77] | 15188009 | *MLH1* | [46,48,76,77] | 22694276; 14993899; 27276710; 15188009 |
| *APC* | [46,48,76,77] | 22694276; 14993899; 27276710; 15188009 | *MMP1* | [46] | 22694276 |
| *ARID1A* | [76] | 27276710 | *MSH2* | [46,48,77] | 22694276; 14993899; 15188009 |
| *AXIN1* | [77] | 15188009 | *MSH6* | [46,48,77] | 22694276; 14993899; 15188009 |
| *BCL-2* | [46] | 22694276 | *MUTYH* | [48,77] | 14993899; 15188009 |
| *BMPR1A* | [46] | 22694276 | *MYC* | [46] | 22694276 |
| *BRAF* | [48,76,77] | 14993899; 27276710; 15188009 | *MYH* | [46] | 22694276 |
| *CTNNB1* | [48,77] | 14993899; 15188009 | *NRAS* | [76] | 27276710 |
| *E2F4* | [46] | 22694276 | *PIK3CA* | [76,77] | 27276710; 15188009 |
| *EP300* | [48,77] | 14993899; 15188009 | *PIK3R1* | [77] | 15188009 |
| *ERBB2* | [76] | 27276710 | *PMS1* | [48,77] | 14993899; 15188009 |
| *FAM123B* | [76] | 27276710 | *PMS2* | [47,48,77] | 22694276; 14993899; 15188009 |
| *FBXW7* | [76,77] | 27276710; 15188009 | *PTEN* | [46] | 22694276 |
| *H2AFZ* | [46] | 22694276 | *RAD21* | [77] | 15188009 |
| *IGF2* | [76] | 27276710 | *RAP1B* | [46] | 22694276 |
| *KRAS* | [76,77] | 27276710; 15188009 | *SMAD2* | [76] | 27276710 |
| *K-ras* | [46] | 22694276 | *SMAD4* | [46,76] | 22694276; 27276710 |
| *KRAS2* | [48] | 14993899 | *SOX9* | [76] | 27276710 |
| *LYN* | [46] | 22694276 | *TACSTD1* | [46] | 22694276 |
| *MADH4* | [48,77] | 14993899; 15188009 | *TBX19* | [46] | 22694276 |
| *MAP2K1* | [77] | 15188009 | *TCF7L2* | [77] | 15188009 |
| *MAP2K4* | [48,77] | 14993899; 15188009 | *TP53* | [46,48,76,77] | 22694276; 14993899; 27276710; 15188009 |
| *MDM2* | [77] | 15188009 | *VEGF* | [46] | 22694276 |
| *MET* | [46] | 22694276 | *VTI1A* | [77] | 15188009 |
